# Genome-Scale Identification of *Legionella pneumophila* Effectors Using a Machine Learning Approach

**DOI:** 10.1371/journal.ppat.1000508

**Published:** 2009-07-10

**Authors:** David Burstein, Tal Zusman, Elena Degtyar, Ram Viner, Gil Segal, Tal Pupko

**Affiliations:** 1 Department of Cell Research and Immunology, George S. Wise Faculty of Life Sciences, Tel Aviv University, Ramat Aviv, Israel; 2 Department of Molecular Microbiology and Biotechnology, George S. Wise Faculty of Life Sciences, Tel Aviv University, Ramat Aviv, Israel; Yale University School of Medicine, United States of America

## Abstract

A large number of highly pathogenic bacteria utilize secretion systems to translocate effector proteins into host cells. Using these effectors, the bacteria subvert host cell processes during infection. *Legionella pneumophila* translocates effectors via the Icm/Dot type-IV secretion system and to date, approximately 100 effectors have been identified by various experimental and computational techniques. Effector identification is a critical first step towards the understanding of the pathogenesis system in *L. pneumophila* as well as in other bacterial pathogens. Here, we formulate the task of effector identification as a classification problem: each *L. pneumophila* open reading frame (ORF) was classified as either effector or not. We computationally defined a set of features that best distinguish effectors from non-effectors. These features cover a wide range of characteristics including taxonomical dispersion, regulatory data, genomic organization, similarity to eukaryotic proteomes and more. Machine learning algorithms utilizing these features were then applied to classify all the ORFs within the *L. pneumophila* genome. Using this approach we were able to predict and experimentally validate 40 new effectors, reaching a success rate of above 90%. Increasing the number of validated effectors to around 140, we were able to gain novel insights into their characteristics. Effectors were found to have low G+C content, supporting the hypothesis that a large number of effectors originate via horizontal gene transfer, probably from their protozoan host. In addition, effectors were found to cluster in specific genomic regions. Finally, we were able to provide a novel description of the C-terminal translocation signal required for effector translocation by the Icm/Dot secretion system. To conclude, we have discovered 40 novel *L. pneumophila* effectors, predicted over a hundred additional highly probable effectors, and shown the applicability of machine learning algorithms for the identification and characterization of bacterial pathogenesis determinants.

## Author Summary

Many pathogenic bacteria exert their function by translocating a set of proteins, termed effectors, into the cytoplasm of their host cell. These effectors subvert various host cell processes for the benefit of the bacteria. Our goal in this study was to identify novel effectors in a genomic scale, towards a better understanding of the molecular mechanisms of bacterial pathogenesis. We developed a computational approach for the detection of new effectors in the intracellular pathogen *Legionella pneumophila*, the causative agent of the Legionnaires' disease, a severe pneumonia-like disease. The novelty of our approach for detecting effectors is the combination of state-of-the-art machine learning classification algorithms with broad biological knowledge on effector biology in a genomic scale. Applying this method, we detected and experimentally validated dozens of new effectors. Notably, our computational predictions had an exceedingly high accuracy of over 90%. In analyzing these effectors we were able to obtain new insights into the molecular mechanism of the pathogenesis system. Our results suggest, for the first time, that over 10% of the *Legionella* genome is dedicated to pathogenesis. Finally, our approach is general and can be utilized to study effectors in many other human pathogens.

## Introduction

A large number of bacterial pathogens utilize secretion systems for pathogenesis. In these systems, a multi-protein complex is used to translocate a repertoire of proteins, termed effectors, into host cells during infection. These effector proteins were found to be critical for the pathogenicity of numerous pathogens, such as *Salmonella enterica*, *Yersinia pestis*, *Pseudomonas syringae* (utilizing a type-III secretion system) [1]–[3], *Legionella pneumophila*, *Coxiella burnetii Helicobacter pylori*, *Bordetella pertussis*, *Agrobacterium tumefaciens*, *Bartonella henselae* (utilizing a type-IV secretion system) [4]–[6], *Vibrio cholerae*, *Mycobacterium tuberculosis*, and *Pseudomonas aeruginosa* (utilizing other secretion systems) [7],[8], making them prime targets for research of bacterial virulence systems.

*L. pneumophila* is an intracellular γ-proteobacteria, which is the causative agent of Legionnaires' disease: a severe pneumonia-like disease in which the bacteria infect and replicate in human alveolar macrophages [9]. *L. pneumophila* also infects a wide range of protozoan hosts, which serve as their environmental reservoir [10],[11]. After internalization by their natural protozoan hosts or by alveolar macrophages, the bacteria are confined to a phagosome and utilize the Icm/Dot type IVb secretion system to subvert host cellular processes [12],[13]. A large number of *L. pneumophila* encoded proteins were found to be translocated into the host cell in an Icm/Dot dependent manner [6], [14]–[16]. Some of the translocated substrates were shown to manipulate host cellular activities, and it is believed that the vast majority of translocated proteins have a functional role during infection.

The first *L. pneumophila* effector, RalF, was identified based on sequence homology to an eukaryotic Guanine Exchange Factor (GEF) domain [17]. Since then, a total of 105 genes were identified as effectors using various approaches such as sequence homology to eukaryotic domains and markers for horizontal gene transfer [17]–[21], interactions with Icm/Dot components [22], transfer of proteins between bacteria [23], genetic assays in yeast [24]–[26], similar regulatory elements [27]–[29], and a predicted secretion signal [30]. Importantly, the cellular function of most effectors is still unknown, and for most of the effectors examined, their knockout failed to reveal an intracellular growth phenotype [20],[22],[26],[29],[31]. Moreover, in some cases, knocking-out a family of paralogous effector-encoding genes simultaneously produced no significant growth defect [23],[32]. These observations suggest the existence of redundancy in terms of effector functionality, or alternatively, some effectors may function only in specific hosts, showing no intracellular growth defect when absent during infection of other hosts.

The *L. pneumophila* Philadelphia-1 genome harbors 3,005 open reading frames (ORFs) [31]. Identifying ORFs that encode for effector proteins is critical for the understanding of the cellular processes involved in *L. pneumophila* pathogenesis. In this study, we developed a novel machine learning approach for the identification of effectors. To the best of our knowledge, this is the first attempt to present the task of effector identification as a computational classification problem. Our approach aims to extract features that distinguish effectors from non-effectors. These features are based, in part, on a systematic review of known characteristics of effectors and in part, on the discovery of novel features. These extracted features were then used to train a variety of machine learning algorithms, which produced a list of predicted effectors sorted by their likelihood. We followed up our predictions with experimental validations, using the CyaA reporter system, which led to the discovery of 40 novel *L. pneumophila* effectors.

## Results

**Figure 1:**
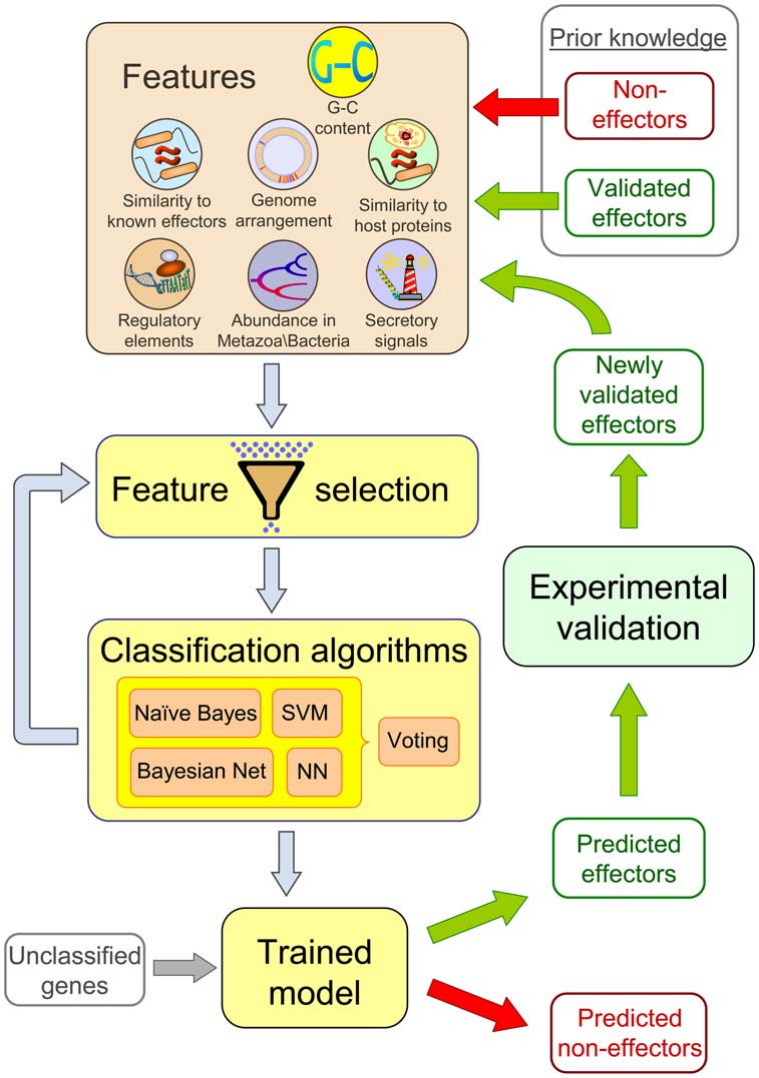
Schematic representation of the computation and experimental steps used for the discovery of novel effectors. A machine learning approach was utilized, in which validated effectors and non-effectors were used as input. Various features expected to separate these two groups were extracted, filtered, and fed into various classifiers. Ten-fold cross validation was used to train the classifiers. The trained classifiers were used to classify the remaining ORFs as either putative effectors or not. High ranking predictions were experimentally validated and the newly validated effectors were used, iteratively, to refine the learning scheme. NN stands for Neural networks and Bayesian Net stands for Bayesian networks.

In this work, we formulated the task of identifying new *L. pneumophila* effectors as a machine learning classification problem. The classification and validation outline is schematically illustrated in Figure 1.

A successful classification is highly dependent on the set of features provided to the learning algorithms. The features we measured cover various attributes that could potentially differentiate effectors from non-effectors. These include genomic attributes, evolutionary based attributes, regulatory network attributes, and attributes specific to the *L. pneumophila* pathogenesis system (Table 1). These features were fed into various machine learning algorithms: support vector machine (SVM), neural network, naïve Bayes, Bayesian networks, and a Voting classifier, which considers all these four classifiers. A feature selection step was applied to each classifier and all classifiers were trained to achieve maximal area under the curve (AUC) based on 10-fold cross validation (as described in Materials and Methods).

**Table 1:** Features used in the machine learning algorithms.

Features	Rationale	References
Sequence similarity to known effector proteins	Effectors were shown to share local sequence similarity	[15], [18], [22]–[24]
Sequence similarity to eukaryotic proteomes	A high number of effectors were shown to contain eukaryotic-like domains	[19],[20],[34],[35]
Taxonomic distribution among Bacteria and Metazoa	Effectors are unlikely to be house keeping genes, which have homologs in numerous other bacteria	
Genome organization	Effector genes cluster in specific genomic regions, possibly as a result of horizontal gene transfer (HGT) events	[23],[29],[32],[33]
G+C content	Effectors were reported to have atypical G+C content, possibly as a result of HGT events	[20]
C-terminal signal	Effectors have a C-terminal secretion signal. Two putative signals were previously suggested	[30],[37],[44]
Regulatory elements	The PmrA and CpxR response regulators regulate numerous effectors	[27],[28]

The output of the best performing classifier is a ranked list of putative effectors, from which we experimentally validated high ranking candidates. Validations were performed by constructing fusion proteins between the putative effector and the catalytic domain of the *Bordetella pertussis* calmodulin dependent CyaA toxin, which converts ATP to cyclic AMP (cAMP). These constructs were expressed in an *L. pneumophila* wild-type strain and in an *icm/dot* mutant, and used for infection of HL-60-derived human macrophages. We consider an effector to be validated if translocation, as determined by the levels of cAMP produced, was observed only in the wild-type strain (see Materials and Methods).

### Effector prediction and validation

We conducted three phases of learning and validation. In each learning phase, we included all the validated effectors known at that time. Specifically, in the second and third phases we added effectors validated in previous phases as well as validated effectors published during the course of this study. Furthermore, the features were updated to reflect the increase in our understanding of effector characteristics and to maximize the information extracted from validated effectors. The effectors discovered in each learning and validation phase are described below.

#### Learning and validation phase 1

**Figure 2:**
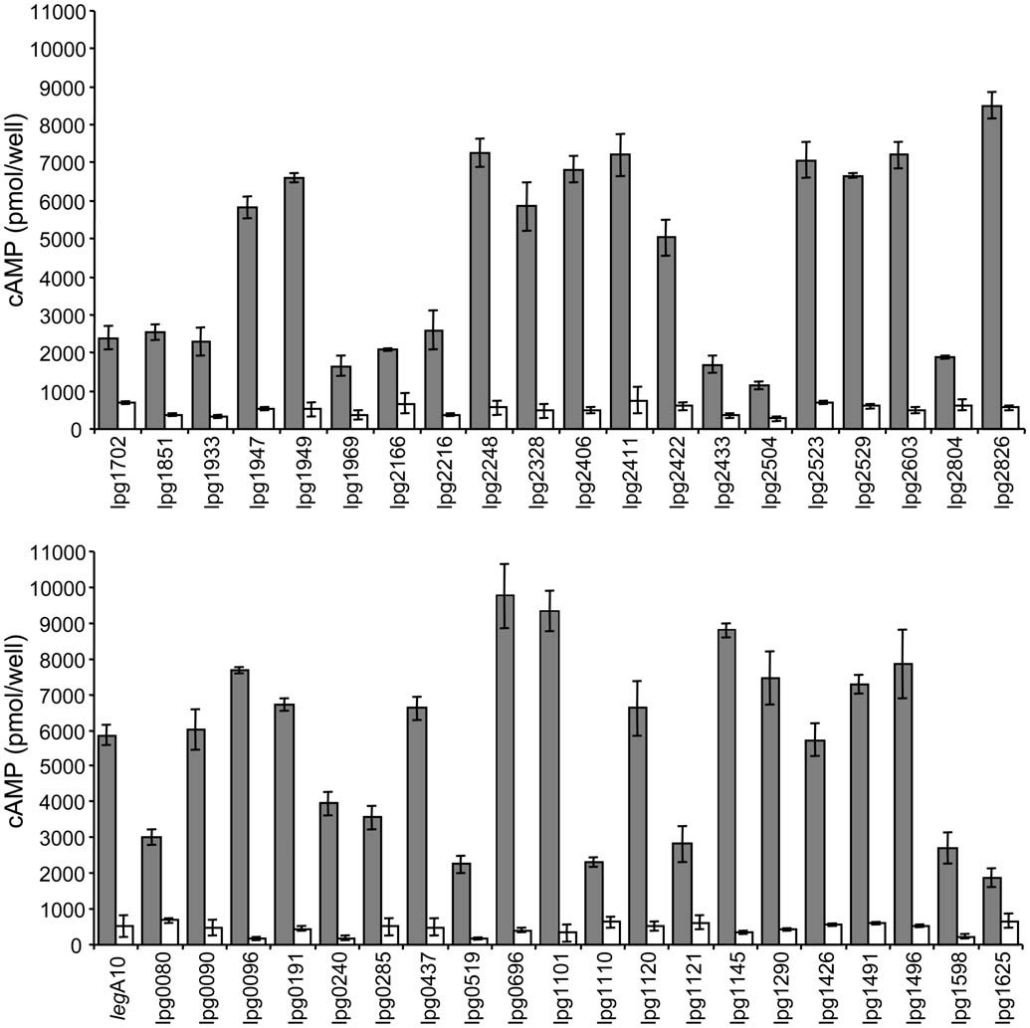
Icm/Dot-dependent translocation of top ranking putative effector proteins. Wild-type strain JR32 (gray bars) and *icmT* mutant GS3011 (white bars) harboring the CyaA fusion proteins (indicated on the left side of the bars) were used to infect HL-60-derived human macrophages, and the cAMP levels of the infected cells were determined (as described in Materials and Methods). The previously validated effector protein LegA10 was used as a positive control [19],[27]. The data are the means for the amount of cAMP per well and the error bars indicate standard deviations of at least 3 independent experiments.

The training set of the first learning phase included 53 known effectors (a list of all the effectors used in each phase appears in Table S1). The Voting classifier outperformed the other learning schemes in cross validation tests, and was hence used to predict novel effectors (see details in Materials and Methods). We decided to focus on the 15 highest ranking predictions. This number was chosen in order to obtain an initial indication regarding the accuracy of the learning scheme. These 15 genes included two *leg* genes that were previously suggested to encode for effectors [20] and the *lirC* gene, which we studied in a parallel project [29]. Table S2 includes the list of the top ranking predictions in each phase, the genes excluded from the analysis, as well as the exact parameters of the best performing classifier. We cloned and expressed the remaining 12 genes and were able to obtain fusion proteins of proper size for 11 of them (Table 2). Next, these 11 fusion proteins were tested for translocation using the CyaA reporter system and were all proven to be genuine effectors (Figure 2 and Table 2).

**Table 2:** *L. pneumophila* putative effectors that were experimentally examined.

Phase	Lpg #^a^	Classification cutoff^b^	Summary^c^
**I**	**lpg0240**, **lpg0437**, **lpg1426**, *lpg1484*, **lpg1496**, **lpg1625**, **lpg1933**, **lpg2216**, **lpg2433**, **lpg2504**, **lpg2523**, **lpg2826**	0.903 (15)	11/11/12
**II**	**lpg0090**, **lpg0191**, *lpg0196*, **lpg0285**, lpg0502, **lpg0519**, **lpg0696**, **lpg1101**, **lpg1120**, **lpg1145**, **lpg1290**, **lpg1491**, **lpg1598**, **lpg1702**, **lpg1851**, **lpg1949**, **lpg1969**, *lpg2206*, **lpg2248**, **lpg2328**, **lpg2411**, **lpg2422**, lpg2507, **lpg2603**	0.998 (50)	21/23/25
**III**	**lpg0080**, **lpg0096**, **lpg1121**, **lpg1947**, **lpg2166**, **lpg2406**, **lpg2529**, **lpg2804**	1 (50)	8/8/8
**Total**		103	40/42/45

a In bold, genes that were validated to encode for effectors; in italics, genes that we failed to clone or express; in plain text, genes that encode proteins that failed to translocate.

b The machine learning cutoff of the classification score used for determining the list of highly confident effectors in each learning phase. In brackets is the number of predicted effectors with equal or higher score than the cutoff value. In total 103 putative effectors had cutoff values similar to those experimentally tested (some of the putative effectors overlap among phases).

c Validated effectors/successfully expressed genes/genes tested.

#### Learning and validation phase 2

The 11 newly validated effectors were added to the training set for the second learning phase and 18 additional effectors, reported during our work [30], were also included. Moreover, a new feature was introduced in this learning phase, which is based on a suggested C-terminal secretion signal [30]. The best performing classifier in this phase was found to be Bayesian networks. Encouraged by the results of the previous phase, we decided to significantly enlarge the number of examined predictions, focusing on the 50 top ranking predictions. These 50 genes included eight *leg* genes, the *lir*C gene, and the *lgt1* gene, which were excluded as they were previously suggested to be effector proteins (see above). Out of the remaining 40 genes, we experimentally examined 25 predictions (Table S2). Twenty three out of these 25 genes were successfully cloned and expressed in *L. pneumophila*. Of these 23 genes, all but two were validated as effectors (Figure 2 and Table 2).

#### Learning and validation phase 3

In the third learning phase we added the above newly validated 21 effectors as well as 31 recently published ones, including the *leg*, *ank*, *ceg*, and *lir* genes [19],[21],[29]. Similar to the second phase, the Bayesian networks classifier achieved the best performance. Fifty top ranking predictions obtained the highest possible score of 1, and we next experimentally tested eight of which in order to obtain a total of 40 new validated effectors. All eight candidates were successfully cloned and proven to be genuine effectors (Figure 2 and Table 2).

#### Summary of validated effectors

In total, we discovered 40 new effectors, which is an addition of 37% to the set of all known *L. pneumophila* effectors. These novel effectors were termed *lem* for *Legionella* effector identified by machine learning. These effectors are listed in Table 3, which additionally includes information regarding homologs in other *L. pneumophila* strains, paralogs within *L. pneumophila* Philadelphia-1, putative regulatory elements, neighboring effectors, and protein sequence motifs.

**Table 3:** Information summary regarding the novel effectors discovered.

ORF	Symbol	Paralogs in *L. pneumophila* Philadelphia-1	Most Proximate effector	PmrA/CpxR^a^	Paris homolog (lpp)	Corby homolog (lpc)	Lens homolog (lpl)	% G+C	Motif^b^
lpg0080	*ceg3*		lpg0081	P	0094			38.3	
lpg0090	*lem1*				0104	0106	0089	37.0	
lpg0096	*ceg4*			P	0110	0115	0096	41.0	
lpg0191	*ceg5*			P	0251			35.0	
lpg0240	*ceg8*			P	0310	0316	0294	35.4	
lpg0285	*lem2*		lpg0284 (*ceg10*)		0361	0362	0337	35.2	
lpg0437	*ceg14*		lpg0436 (*legA11*)	P+C	0504	2905	0480	36.5	
lpg0519	*ceg17*		lpg0518	P				36.4	
lpg0696	*lem3*		lpg0695 (*legA8*)	C	0751	2598	0733	36.7	
lpg1101	*lem4*				1101	2154	1100	34.3	
lpg1110	*lem5*				1111	2142	1114	35.2	
lpg1120	*lem6*	lpg2433 (*ceg30*)	lpg1121 (*ceg19*)			2043		31.9	
lpg1121	*ceg19*		lpg1120 (*lem6*)	P	1121	0578	1126	38.0	
lpg1145	*lem7*		lpg1144 (*cegC3*)		1147	0608	1151	36.1	
lpg1290	*lem8*				1253			36.1	
lpg1426	*vpdC*	lpg2410 (*vpdA*)			1381	0842	1377	35.0	PL
lpg1491	*lem9*		lpg1488 (*legC5*)		1447			32.7	
lpg1496	*lem10*		lpg1491 (*lem10*)		1453	0915	1530	36.4	
lpg1598	*lem11*		lpg1602 (*legL2*)		1556	1025	1427	31.2	
lpg1625	*lem12*		lpg1621 (*ceg23*)		1595	1052	1398	33.8	
lpg1702	*lem13*		lpg1701 (*legC3*)		1667	1131	1661	37.5	CC
lpg1851	*lem14*				1818	1296	1817	36.2	
lpg1933	*lem15*	lpg2400(*legL7*)			1914	1406	1903	35.9	
lpg1947	*lem16*		lpg1948 (*legLC4*)		1930			32.1	CC
lpg1949	*lem17*		lpg1948 (*legLC4*)		1931	1422	1918	36.1	
lpg1969	*lem18*		lpg1966 (*lirF*)		1952	1452	1941	37.2	CC
lpg2166	*lem19*				2104	1626	2093	35.5	CC
lpg2216	*lem20*		lpg2215 (*legA2*)	P	2167	1681	2141	35.1	CC
lpg2248	*lem21*				2202	1717	2174	38.8	
lpg2328	*lem22*		lpg2327		2276	1795	2248	38.0	
lpg2406	*lem23*		lpg2407		2472	2070	2329	37.6	
lpg2411	*lem24*		lpg2410 (*vpdA*)		2480	2064	2335	32.9	
lpg2422	*lem25*				2487	2055	2345	37.9	CC
lpg2433	*ceg30*	lpg0126 (*cegC2*)		P	2500	2043	2353	37.9	
lpg2504	*ceg32*		lpg2508 (*sdjA*)	P	2572	1967	2426	34.6	
lpg2523	*lem26*		lpg2527					36.6	
lpg2529	*lem27*		lpg2527		2594	1942	2449	38.0	
lpg2603	*lem28*				2656	0539	2526	35.3	
lpg2804	*lem29*			P+C	2850	3090	2719	38.2	
lpg2826	*ceg34*		lpg2829 (*sidH*)	P		3113	2741	34.3	ANK

a P: contains PmrA regulatory element; C: contains CpxR regulatory element.

b PL: Phospholipase; CC: Coiled-coil; ANK: Ankyrin-repeat.

#### Predicted effectors

The high accuracy of our predictions (above 90%) suggests that a large number of the top ranking predictions of the third learning phase that were not experimentally tested are effectors. Specifically, the 126 highest ranking predictions, for which the confidence score is higher than 0.995, are listed in Table S3.

#### The ability of each feature group to distinguish effectors from non-effectors

The features used as input to the machine learning classifiers described above are divided into seven groups (as described in Table 1). We have tested the classification performance, when the learning was limited to a single group at a time in order to underline the features that best characterize effectors (Table 4). The feature group with the highest classification performance was found to be “Taxonomic distribution among Bacteria and Metazoa”, which correctly classified 89.9% of the ORFs (Table 4). The second and third top ranking feature groups are based on homology to known effector proteins and to eukaryotic proteins, with correct classification rates of 80.2% and 78.7%, respectively. Notably, these three top ranking feature groups are all based on protein sequence similarity. Also noteworthy is the ability of the C-terminal signal feature group to correctly classify only 60.1% of the ORFs. This indicates the importance of a more detailed understanding of this signal (see below). Importantly, combining all feature groups together resulted in prediction accuracy of above 95% on the training set, indicating that combining various sources of information enables maximal accuracy.

**Table 4:** Classification performance of each feature group separately.

Feature group	Correct rate	AUC
Taxonomic distribution among Bacteria and Metazoa	89.9%	0.96
Sequence similarity to known effector proteins	80.2%	0.78
Sequence similarity to eukaryotic proteomes	78.7%	0.78
G+C content	78.4%	0.78
Genome organization	74.3%	0.77
Regulatory elements	70.9%	0.71
C-terminal signal	60.1%	0.6
All features combined	95.9%	0.98

### Computational characterization of 145 *L. pneumophila* effectors

Thus far, analyses of attributes characterizing effectors were based on a limited set of a few dozens of validated effectors [20], [23], [28]–[30]. The availability of 145 validated effectors motivated us to perform an in-depth analysis of effector characteristics. Interesting observations were obtained regarding three such characteristics – distribution of effectors in the genome, G+C content of effector encoding genes, and the C-terminal secretion signal.

#### Distribution of effectors in the genome

**Figure 3:**
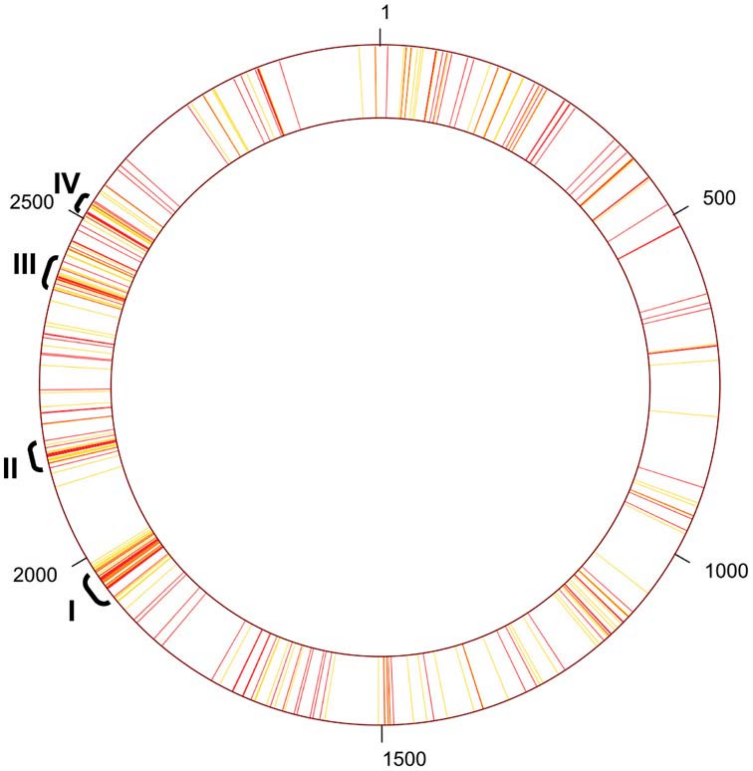
Schematic representation of the distribution of effectors and putative effectors in the *L. pneumophila* genome. Validated effectors are in red and putative effectors are in yellow. Roman digits indicate genomic regions enriched with effector encoding genes (as described in Table 5). Numbers represent lpg (*L. pneumophila* Philadelphia-1 gene) identifier. Notably, the units used for this schematic presentation are ORFs rather than base-pairs.

Previous studies have discovered genomic regions that are enriched with effector encoding genes [23],[29],[33]. These observations suggest that effectors cluster in the genome non-randomly. Figure 3 presents the distribution of all validated effectors (in red) as well as the distribution of the 126 top ranking predicted effectors (in yellow). This spatial distribution of effector-encoding genes significantly differs from a uniform one (Wald-Wolfowitz test of randomness; *p-value*<10^−23^). Interestingly, including the 126 putative effectors increases the clustering signal, providing an additional support for the clustering of effectors (Wald-Wolfowitz; *p-value*<10^−45^). Our analysis suggests four genomic regions (I–IV) highly enriched with effector encoding genes (Figure 3; Table 5). The observed effector clusters defined by us match genomic regions previously described: region I includes *lir* genes and several other effectors [29], regions II and IV include numerous *sid* genes [23],[33], and region III was described before as a hyper-variable region [29].

**Table 5:** Genomic regions enriched with effector encoding genes.

Region^a^	Validated effectors	Predicted effectors	G+C^b^
**I**lpg1933–lpg1978 (46/16/7)	lpg1933 (*lem15*), lpg1947 (*lem16*), lpg1948 (*legLC4*), lpg1949 (*lem17*), lpg1950 (*ralF*), lpg1953 (*legC4*), lpg1958 (*legL5*), lpg1960 (*lirA*), lpg1962 (*lirB*), lpg1963 (*lirC*), lpg1964(*lirD*), lpg1965 (*lirE*), lpg1966 (*lirF*), lpg1969 (*lem18*), lpg1976 (*legG1*), lpg1978 (*setA*)	lpg1952, lpg1957, lpg1959, lpg1961, lpg1968, lpg1972, lpg1975	36.8%
**II**lpg2137–lpg2176 (40/9/8)	lpg2137 (*legK2*), lpg2144 (*legAU13/ceg27/ankB*), lpg2153 (*sdeC*), lpg2154 (*sde*), lpg2155 (*sidJ*), lpg2156 (*sdeB*), lpg2157 (*sdeA*), lpg2166 (*lem19*), lpg2176 (*legS2*),	lpg2143, lpg2147, lpg2148, lpg2149, lpg2150, lpg2159, lpg2160, lpg2170	38.1%
**III**lpg2391–lpg2433 (43/9/8)	lpg2391 (*sdbC*), lpg2400 (*legL7*), lpg2406 (*lem23*), lpg2407, lpg2409 (*ceg29*), lpg2410 (*vpdA*), lpg2411 (*lem24*), lpg2422 (*lem25*), lpg2433 (*ceg30*)	lpg2395, lpg2403, lpg2408, lpg2413, lpg2414, lpg2416, lpg2424, lpg2425	38.4%
**IV**lpg2504–lpg2529 (26/8/6)	lpg2504 (*ceg32*), lpg2508 (*sdjA*), lpg2509 (*sdeD*), lpg2510 (*sdcA*), lpg2511 (*sidC*), lpg2523 (*lem26*), lpg2527, lpg2529 (*lem27*)	lpg2505, lpg2518, lpg2519, lpg2520, lpg2522, lpg2525	37.6%

a Number of ORFs in region/validated effectors/predicted effectors.

b G+C content of coding regions.

#### G+C content of effector encoding genes

It was previously suggested that a large number of effectors are of eukaryotic origin [19],[20],[34],[35]. Such putative horizontally transferred genes are often characterized by atypical G+C content. Here, we statistically compared the G+C content of effector encoding genes to the remaining ORFs. Effectors were found to have a relatively low G+C content: the average G+C content of effector encoding genes was 36.9% compared with a 39.3% G+C content of the rest of the coding sequence (two tailed *t*-test, *p-value*<10^−15^). These results were also found to be valid when analyzing G+C content in other *L. pneumophila* strains. In the Paris, Lens, and Corby coding sequences, the average G+C content of effectors was 37.1%, 37.2%, and 37.2%, while the G+C content of the rest of the coding sequence was 38.8%, 38.8%, and 38.6%, respectively. These results are all statistically significant (*p-value*<10^−11^, *p-value*<10^−10^, and *p-value*<10^−7^, respectively). These results are in agreement with a previous report, in which lower G+C content was found in the *leg* genes [20]. Furthermore, the G+C content of the 126 putative effectors was also significantly lower than the overall G+C content. Notably, *Tetrahymena thermophila*, a known host of *Legionella*, has a very low G+C content of 27% [36]. Taken together, our results support the hypothesis that horizontal gene transfer (HGT) serves as a major mechanism for acquiring effectors-encoding genes probably from the protozoan hosts.

#### The C-terminal secretion signal

While it is evident that a signal for effector translocation via the Icm/Dot secretion system resides in the effectors' C-terminal, the exact identity of this signal remains an open question. It has been suggested that a hydrophobic residue or a proline residue at the −3 or −4 position, relative to the C-terminal amino acid, is critical for effector translocation [37]. The characterization of the C-terminal signal was further extended suggesting that in addition to the hydrophobic residue, the C-terminus of effectors is enriched with tiny, polar, and charged amino acids [30]. However, these analyses were based on a relatively limited set of validated effectors.

**Figure 4:**
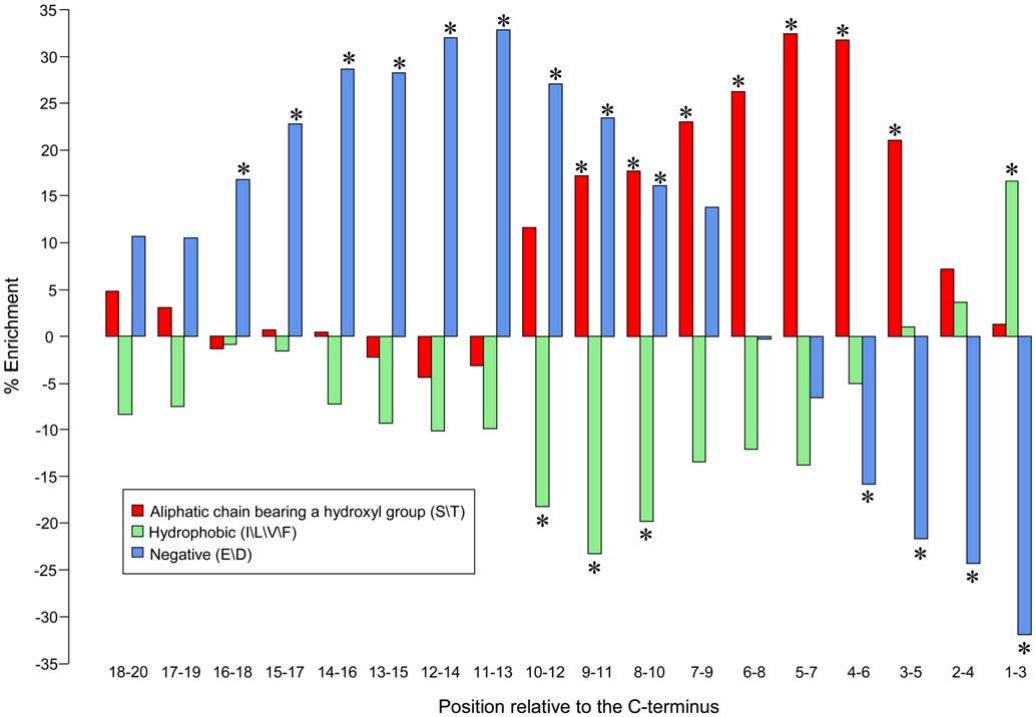
The putative secretion signal at the C-terminus of effectors. The enrichment and depletion pattern of groups of amino acids within the 20 C-terminal residues of effectors is shown. Amino acids with aliphatic side-chains bearing a hydroxyl group (S/T) are in red, hydrophobic amino acids (I/L/V/F) in green, and negatively charged amino acids (E/D) in blue. Statistically significant enrichments or depletions (*G*-test; *p-value*<0.01 after Bonferroni correction) are marked with asterisks.

We searched for the C-terminal signal across all the 145 validated effectors, focusing on the 20 C-terminal positions. First, we analyzed for each position each of the 20 amino acids and compared their frequencies in effectors to their frequencies in non-effectors (altogether 20×20 = 400 tests). Of these 400 tests, 17 were significant (*G*-test; *p-value*<0.05, after Bonferroni correction; Table S4). In 16 of these 17 significant tests, the amino acids were aspartic acid, glutamic acid, serine, and threonine. This suggests two groups with similar physicochemical properties: the first two are negative amino acids and the latter two have aliphatic side chains bearing a hydroxyl group. We thus repeated the enrichment/depletion test with respect to physico-chemical groups of amino acids: positively charged (lysine and arginine), negatively charged (aspartic acid and glutamic acid), an aliphatic side-chain bearing a hydroxyl group (serine and threonine), and hydrophobic (leucine, isoleucine, valine, and phenylalanine). In addition, a sliding window approach was used to allow flexibility in the position of the signal with respect to the protein C-terminus. The differences in abundance of these amino-acid groups between effectors and non-effectors using a sliding window of three amino acids are presented in Figure 4.

Our analyses suggest extensive depletion of negative amino acids in positions −1 to −6 and considerable enrichment of these amino acids in positions −8 to −18; serine and threonine are enriched in positions −3 to −11; hydrophobic amino acids are enriched in positions −1 to −3 and depleted in positions −8 to −12 (all these results are statistically significant using *G*-test with *p-values*<0.01 after Bonferroni correction).

## Discussion

In this study we have identified 40 new effectors, bringing the total of known effectors to 145. The high rate of correct predictions suggests that effectors can indeed be clearly distinguished from the remaining ORFs according to the features described in this work. According to available expression array data [38], these newly discovered effectors are expressed during intracellular growth in amoeba. The expression of 18 effectors was elevated post infection, the expression of 7 effectors was decreased, and the remaining 15 did not change substantially (less than 1.5 fold change). The functional role of these effectors during infection has yet to be determined.

Regarding the evolutionary origin of effectors, the low G+C content and spatial clustering (Figure 3) support the hypothesis that effectors are often transferred via HGT. These results are in agreement with a recent publication showing that two *L. pneumophila* effectors were most likely acquired from Protozoa [39], and with an additional evolutionary study, in which HGT from an amoeba to *Legionella* was demonstrated [40]. Regarding our 40 newly discovered effectors, the homology to eukaryotes was found to be restricted to specific domains, thus, it is currently impossible to pinpoint the exact evolutionary origins for these genes.

The 145 currently validated effectors make *L. pneumophila* the organism with the highest number of validated effectors. This can provide a lower bound on the percentage of *L. pneumophila* ORFs that encode for effector proteins – 5% of the total number of ORFs. However, assuming that a large fraction of our predicted effectors are genuine ones, the estimate becomes close to 10% (about 300 effectors), which constitutes an exceedingly large pool of effectors relative to any other known pathogenesis related secretion systems.

An important result of our study is the list of additional 126 predicted effectors. Support for the validity of these putative effectors comes from a recent study concerning yeast growth defect of *L. pneumophila* ORFs [25]. In this study, three new effectors were validated. One of them (*ceg19*) was independently validated in our study, and an additional ORF (*ceg9*) is included in our list of putative effectors (Table S2). In the same study, 12 additional ORFs that showed yeast growth defects and were not experimentally tested for translocation were included in our list of putative effectors. Since effectors were shown to often confer a yeast growth defect phenotype [41],[42], this provides additional support for the validity of these putative effectors. Notably, out of our list of 126 predicted effectors, an additional effector (*legK1*) was also recently validated, see Table S2 [43].

Two previous studies characterized the secretion signal located at the effector C-terminus [30],[37]. We utilized the high number of validated effectors to statistically analyze the abundance of amino-acid groups at the C-terminus of effectors versus non-effectors and suggested a detailed description of this signal (Figure 4). We further computed the secretion signal among the 126 predicted effectors. The resulting signal is essentially similar to the one inferred from the list of validated effectors (Figure S1). This similarity supports both the validity of the putative effectors as well as the biological significance of the secretion signal. It should be noted that the previously suggested secretion signals [30],[44] were not retained by the classification algorithms in the final learning phase and hence, detecting the secretion signal defined in this work among the putative effectors cannot be attributed to the inclusion of these features when training the classifier.

Our machine learning approach is general and thus can be applied to other pathogens. However, the applicability of this approach to other bacteria requires a set of validated effectors, adjustment of the features in order to optimally discriminate these effectors from non-effectors, and an experimental system for prediction validation. Nevertheless, we anticipate that the overall scheme of effector identification will be useful for many pathogenesis systems, when their effector research reaches a proper stage. The pathogenesis system most resembling the one of *L. pneumophila* is that of *C. burnetii*, an obligate intracellular pathogen and a potential bioterrorism agent. These bacteria utilize an Icm/Dot type-IVb secretion system and translocate effector proteins in a mechanism similar to that of *L. pneumophila*, as indicated by the ability of *C. burnetii* effectors to translocate via the *L. pneumophila* translocation system [21]. Most of our features, the learning algorithms, and the experimental validation experiments used in this study to identify effectors are applicable with relatively minor changes to the study of *C. burnetii* pathogenesis, while for more distantly related pathogens (e.g., those using type-IVa secretion systems) further adjustments are required.

To summarize, in this work we have developed a combined computational-experimental approach to identify and validate pathogenesis determinants on a genomic scale. We have increased the number of validated effectors by more than 37% and suggested over a hundred putative ones. We have developed a general machine learning scheme for the prediction of effectors, which can be updated when new information becomes available. Finally, this work suggests that our approach is applicable in the identification and characterization of effectors in other bacterial pathogenesis systems.

## Materials and Methods

### Sequences

The following genomes were acquired from the RefSeq database at NCBI (http://www.ncbi.nlm.nih.gov/RefSeq/): *L. pneumophila* Philadelphia-1 (NC_002942); *L. pneumophila* Lens (NC_006366 and NC_006369); *L. pneumophila* Corby (NC_009494); *L. pneumophila* Paris (NC_006365 and NC_006368); *Escherichia coli* strain K-12 DH10B (NC_010473), and *Pseudomonas fluorescens* Pf-5 (NC_004129). Two datasets of all human proteins were downloaded from ftp://ftp.ncbi.nih.gov/genomes/H_sapiens/protein/. The first includes all the human annotated proteins and the second is comprised of *ab initio* protein predictions of “Gnomon”, an NCBI eukaryotic gene prediction tool (http://www.ncbi.nlm.nih.gov/genome/guide/gnomon.shtml). The genome of *Dictyostelium discoideum* was downloaded from DictyBase (http://dictybase.org/). The *T. thermophila* genome was acquired from the TIGR database (http://www.tigr.org/tdb/e2k1/ttg/).

### Effector and non-effector datasets

For each of the three learning phases (see Results), we constructed a dataset of known effectors and a dataset of non-effectors. The size of each non-effector dataset was five folds larger than its corresponding effector dataset. For each learning phase the effector dataset included all effectors known at that time (published effectors and effectors we validated at previous learning phases). Since a dataset of experimentally validated non-effectors is unavailable, we searched for genes that are present in both *L. pneumophila* and *E. coli*, under the premise that such genes are most likely not related to the pathogenicity of *L. pneumophila* and are thus expected to be non-effectors. Specifically, BLAST-P similarity scores were computed for each *L. pneumophila* protein against the proteins of *E. coli*. An *L. pneumophila* protein was defined as a non-effector if it has a hit from *E. coli* with an E-value lower than 10^−20^ and sequence similarity higher than 50%. Additionally, the proteins that constitute the Icm/Dot secretion system were included in the non-effector datasets. A full list of all the effectors and non-effectors used in each phase is given in Table S1.

### Features and feature selection

Each ORF in the *L. pneumophila* Philadelphia-1 genome was described using a vector of features. The features used for each learning phase are detailed in Table S2 and are summarized in Table 1.

#### Sequence similarity to known effector proteins

Two features based on local sequence similarity to effector proteins were measured. Both of these features rely on all-against-all searches of *L. pneumophila* Philadelphia-1 proteins using local BLAST-P [45]. The first is the BLAST bit score to the most similar known effector. The second is the number of known effectors that have significant similarity (with E-value<0.01) to the ORF in question.

#### Sequence similarity to eukaryotic proteomes

For each *L. pneumophila*'s ORF, four features based on sequence similarity to host proteomes were defined. The proteomes analyzed for this task were from two protozoans: *T. thermophila* and *D. discoideum*, from *Homo sapiens*, and from a human protein dataset of NCBI's Gnomon *ab initio* protein predictions. Specifically, each of the four features is defined as the highest BLAST-P bit score against each of the abovementioned protein datasets.

#### Taxonomic distribution among Bacteria and Metazoa

While the above features are based on sequence similarity to specific genomes (hosts and *L. pneumophila*), two additional features were based on the overall taxonomical distribution of each ORF. The first feature counts the number of homologous proteins in the entire bacterial domain. Similarly, the second feature counts the number of homologs among Metazoa. Specifically, the number of homologs is defined as the number of hits with a bit score higher than 100 against NCBI's protein non-redundant (nr) database. These numbers were extracted from the taxonomic grouping information of Blink (http://www.ncbi.nlm.nih.gov/Web/Newsltr/Spring04/blink.html).

#### Genome organization

The genomic distance between a given ORF and the closest effector in the genome was measured. A distance of *i* indicates that the ORF and the closest known effector are separated by *i*-1 ORFs that are not annotated as effectors. We preferred to measure distance in ORF units, rather than in base-pairs to eliminate bias of long genes and to focus on gene organization. In addition, the number of effectors in the genomic vicinity of each ORF was recorded. Specifically, for any 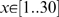, the number of known effectors residing *x* ORFs upstream and downstream was measured.

#### G+C content

The G+C content of each ORF was measured as the fraction of G and C out of the ORF length in base pairs.

#### C-terminal signal

The number of occurrences of the motif suggested as a secretion signal by Hohlfeld et al. [44] was used as a feature. The motif, which resides in the 20 C-terminal amino-acid positions, consists of two positively charged amino acids, separated by three or four amino acids, out of which at least one is negatively charged. An additional feature was based on the secretion signal suggested by Kubori et al. [30], which includes frequent tiny (alanine, glycine, serine, threonine) and polar (glutamine, aspartic acid, glutamic acid, histidine, lysine, asparagine, arginine, serine, threonine) amino acids in the C-terminal amino-acid positions of the protein. The feature we considered is the fraction of tiny and polar amino acids within the 14 C-terminal positions.

#### Regulatory elements

Two features were based on the existence of conserved regulatory elements: one for the response regulator PmrA and one for CpxR. Position specific score matrices (PSSMs) [46] for each of these regulatory elements were constructed, and a single pseudo-count was added to each position. The PSSMs were based on a set of genes that were shown to be regulated by these elements [27],[28] and are given as Table S4. These PSSMs were used to search for putative regulatory elements between −200 bp to +50 bp relative to the first nucleotide of the start codon. The score of the best match to each PSSM was recorded. Notably, since CpxR can recognize its target sequence in the reverse strand as well, the highest score was sought on both strands.

### Machine learning algorithms

Machine learning algorithms were performed using the WEKA package [47]. The following classification algorithms were tested: Naïve Bayes, Bayesian networks, SVM (SMO), Neural networks (Multilayer perceptron), and a Voting algorithm that is based on these four algorithms. Feature selection was performed using a “Wrapper” to find the best performing features for each one of the algorithms, using hill-climbing search algorithms. The classifiers were trained on datasets in which the ratio of effectors to non-effectors was 1∶5. Specifically, as more effectors were included in the second and third phases, the number of non-effectors was increased accordingly to maintain this ratio.

The classification performance was evaluated on the train datasets for each classifier separately. Classification performance for each classifier was evaluated using 10-fold cross validation, i.e., 90% of the training data were randomly chosen and used to tune the parameters of each classifier, and the remaining 10% were used to evaluate the classifier performance [47]. The performance score is measured in terms of AUC, which accounts for both the fraction of true positives (correctly classified effectors) and false positives (ORFs erroneously classified as effectors). Since the performance depends on the division of the training data, the procedure is repeated 10 times, so that each 10% is used once to evaluate performance. Classifier accuracy is defined as the average over these 10 repeats. The classifier with the highest average AUC was used at each learning phase to predict effectors.

When we evaluated the classification performance of each feature group separately, a dataset in which the ratio of 1∶1 between effectors and non-effectors was used. This was done to avoid artificial high performance stemming from the excess of non-effectors in the training data.

The computer code used to implement the machine learning scheme described here is available in http://www.tau.ac.il/~talp/LegionellaMachineLearning.

### Bacterial strains and media

The *L. pneumophila* strains used in this study were *L. pneumophila* JR32, a streptomycin-resistant, restriction-negative mutant of *L. pneumophila* Philadelphia-1, which is a wild-type strain in terms of intracellular growth [48] and GS3011 an *icm*T deletion mutant [49]. The *E. coli* strain used was MC1022 [50]. Bacterial media, plates, and antibiotic concentrations were used as described previously [51].

### Construction of *cyaA* fusions

The plasmid pMMB-cyaA-C [28] was used for the cloning of all the *cya*A fusions constructed. All the genes examined were amplified by PCR using a pair of primers containing suitable restriction sites at the 5′ end. The PCR products were subsequently digested with the relevant enzymes, and cloned into the pMMB-cyaA-C vector to generate plasmids. Table S5 includes the pair of primers, the enzymes used for digestion, and the generated plasmids. The generated plasmids were sequenced to verify that no mutations were introduced during the PCR. Furthermore, the formation of a fusion protein with a proper size was validated by Western analysis using the CyaA antibody 3D1 (Santa Cruz Biotechnology, Inc.).

### CyaA translocation assay

Others and we have utilized the CyaA translocation assay to validate effector proteins [17],[27],[28],[30],[33]. Specifically, differentiated HL-60-derived human macrophages plated in 24-well tissue culture dishes at a concentration of 2.5×10^6^ cells/well were used for the assay. Bacteria were grown on ABCYE (ACES buffered charcoal yeast extract) plates containing chloramphenicol (Cm) for 48 h. The bacteria were scraped off the plate, calibrated to OD_600_ of 0.2, and 20 µl of these bacteria were spotted on an ABCYE plate containing Cm and 1 mM isopropyl-ß-D-thiogalactopyranoside (IPTG) and grown for 20 h. The bacteria were then scraped off the plate and calibrated in order to result with a multiplicity of infection (MOI) of 5 during infection. Cells were infected with bacteria harboring the appropriate plasmids and the plates were centrifuged at 180 *g* for 5 minutes followed by incubation at 37°C under CO_2_ (5%) for 2 h. Cells were then washed twice with ice-cold PBS buffer (1.4 M NaCl, 27 mM KCl, 100 mM Na_2_HPO_4_, 18 mM KH_2_PO_4_) and lysed with 200 µl of lysis buffer (50 mM HCl and 0.1% Triton X-100) at 4°C for 30 minutes. Lysed samples were boiled for 5 minutes, centrifuged for 10 minutes, and the supernatants were neutralized with NaOH. The levels of cAMP were determined using the cAMP Biotrak enzyme immunoassay (EIA) system (GE-healthcare) according to the manufacturer's instructions. The presence of the CyaA fusion proteins was detected by Western blot, using monoclonal antibody anti-CyaA 3D1 (Santa Cruz Biotechnology, Inc.) diluted 1∶500 and goat anti-mouse IgG conjugated to HRP (Jackson Immunoresearch Laboratories, Inc.) diluted 1∶10,000.

## Supporting Information

Figure S1 (0.03 MB PDF)

Table S1 (0.06 MB PDF)

Table S2 (0.10 MB PDF)

Table S3 (0.05 MB PDF)

Table S4 (0.05 MB PDF)

Table S5 (0.03 MB PDF)

Table S6 (0.06 MB PDF)
