# Supplementary figures and images for: Genome-Scale Identification of Legionella pneumophila Effectors Using a Machine Learning Approach
Source: PLoS Pathog. 2009 Jul 10;5(7):e1000508. doi: 10.1371/journal.ppat.1000508 (PMC2701608; doi:10.1371/journal.ppat.1000508)

Burstein et al, Supplemental Figure S1

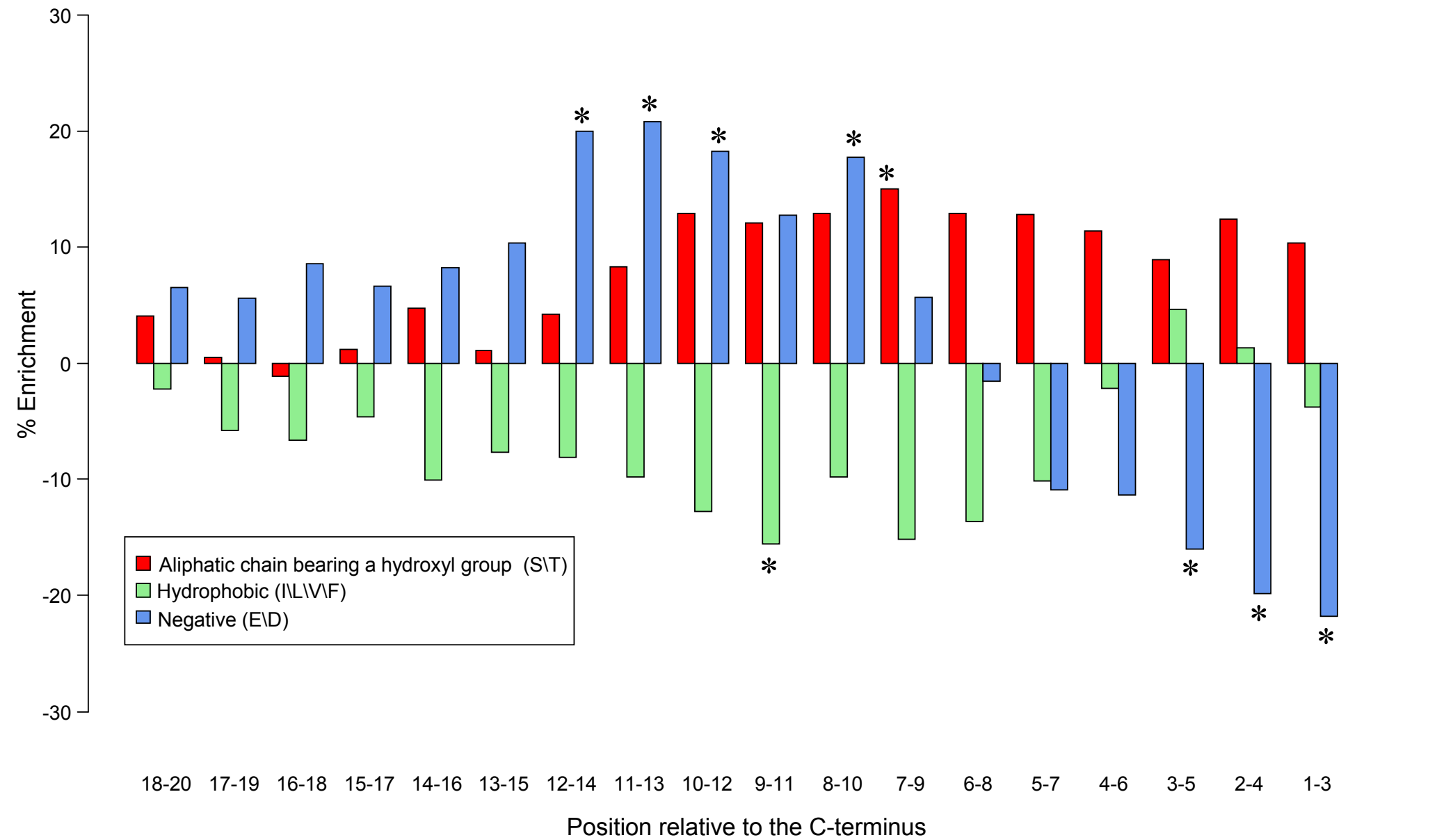

Supplement: Figure S1 — (0.03 MB PDF) [file ppat.1000508.s001.pdf]
