# Supplementary material for: Genome-Scale Identification of Legionella pneumophila Effectors Using a Machine Learning Approach
Source: PLoS Pathog. 2009 Jul 10;5(7):e1000508. doi: 10.1371/journal.ppat.1000508 (PMC2701608; doi:10.1371/journal.ppat.1000508)
Supplement: Table S1 — (0.06 MB PDF) [file ppat.1000508.s002.pdf]

Phase			Training set										
1st	effectors			non effectors									
	#	lpg	Symbol	#	lpg	#	lpg	#	lpg	#	lpg	#	lpg
	1	lpg1950	*ralF*	1	lpg0001	54	lpg0450	107	lpg0818	160	lpg1591	213	lpg2636
	2	lpg2793	*lepA*	2	lpg0004	55	lpg0451	108	lpg0835	161	lpg1592	214	lpg2646
	3	lpg2490	*lepB*	3	lpg0009	56	lpg0452	109	lpg0836	162	lpg1690	215	lpg2649
	4	lpg0940	*lidA*	4	lpg0052	57	lpg0453	110	lpg0841	163	lpg1711	216	lpg2650
	5	lpg2718	*wipA*	5	lpg0072	58	lpg0454	111	lpg0842	164	lpg1712	217	lpg2654
	6	lpg0642	*wipB*	6	lpg0094	59	lpg0455	112	lpg0847	165	lpg1714	218	lpg2667
	7	lpg2298	*ylfA/legC7*	7	lpg0097	60	lpg0456	113	lpg0872	166	lpg1719	219	lpg2669
	8	lpg1884	*ylfB/legC2*	8	lpg0117	61	lpg0458	114	lpg0888	167	lpg1722	220	lpg2670
	9	lpg0284	*ceg10*	9	lpg0125	62	lpg0459	115	lpg0932	168	lpg1723	221	lpg2674
	10	lpg1621	*ceg23*	10	lpg0137	63	lpg0462	116	lpg0942	169	lpg1724	222	lpg2675
	11	lpg2409	*ceg29*	11	lpg0139	64	lpg0476	117	lpg0946	170	lpg1746	223	lpg2676
	12	lpg0898	*ceg18*	12	lpg0157	65	lpg0479	118	lpg1131	171	lpg1748	224	lpg2686
	13	lpg2591	*ceg33*	13	lpg0218	66	lpg0486	119	lpg1181	172	lpg1750	225	lpg2687
	14	lpg0227	*ceg7*	14	lpg0314	67	lpg0513	120	lpg1194	173	lpg1757	226	lpg2688
	15	lpg0012	*cegC1*	15	lpg0317	68	lpg0525	121	lpg1202	174	lpg1766	227	lpg2689
	16	lpg0126	*cegC2*	16	lpg0318	69	lpg0530	122	lpg1215	175	lpg1767	228	lpg2702
	17	lpg1144	*cegC3*	17	lpg0319	70	lpg0531	123	lpg1282	176	lpg1770	229	lpg2706
	18	lpg2200	*cegC4*	18	lpg0321	71	lpg0533	124	lpg1284	177	lpg1777	230	lpg2707
	19	lpg0038	*legA10/ankQ*	19	lpg0322	72	lpg0534	125	lpg1286	178	lpg1778	231	lpg2709
	20	lpg0436	*legA11/ankJ*	20	lpg0323	73	lpg0535	126	lpg1297	179	lpg1791	232	lpg2712
	21	lpg1488	*legC5*	21	lpg0324	74	lpg0543	127	lpg1304	180	lpg1801	233	lpg2713
	22	lpg1660	*legL3*	22	lpg0326	75	lpg0548	128	lpg1306	181	lpg1840	234	lpg2714
	23	lpg1958	*legL5*	23	lpg0327	76	lpg0552	129	lpg1334	182	lpg1842	235	lpg2741
	24	lpg2400	*legL7*	24	lpg0328	77	lpg0577	130	lpg1341	183	lpg1853	236	lpg2764
	25	lpg1948	*legLC4*	25	lpg0329	78	lpg0578	131	lpg1362	184	lpg1854	237	lpg2765
	26	lpg1890	*legLC8*	26	lpg0332	79	lpg0592	132	lpg1364	185	lpg1858	238	lpg2768
	27	lpg0621	*sidA*	27	lpg0334	80	lpg0601	133	lpg1369	186	lpg1859	239	lpg2773
	28	lpg1642	*sidB*	28	lpg0335	81	lpg0602	134	lpg1373	187	lpg1860	240	lpg2796
	29	lpg2511	*sidC*	29	lpg0336	82	lpg0636	135	lpg1383	188	lpg1861	241	lpg2797
	30	lpg2465	*sidD*	30	lpg0338	83	lpg0639	136	lpg1388	189	lpg1872	242	lpg2810
	31	lpg0234	*sidE*	31	lpg0339	84	lpg0640	137	lpg1395	190	lpg1906	243	lpg2812
	32	lpg2584	*sidF*	32	lpg0341	85	lpg0641	138	lpg1396	191	lpg1927	244	lpg2822
	33	lpg1355	*sidG*	33	lpg0349	86	lpg0651	139	lpg1397	192	lpg2012	245	lpg2833
	34	lpg2829	*sidH*	34	lpg0353	87	lpg0659	140	lpg1415	193	lpg2022	246	lpg2834
	35	lpg0275	*sdbA*	35	lpg0354	88	lpg0685	141	lpg1417	194	lpg2025	247	lpg2839
	36	lpg2482	*sdbB*	36	lpg0355	89	lpg0688	142	lpg1421	195	lpg2028	248	lpg2868
	37	lpg2391	*sdbC*	37	lpg0384	90	lpg0691	143	lpg1425	196	lpg2189	249	lpg2872
	38	lpg2510	*sdcA*	38	lpg0388	91	lpg0694	144	lpg1443	197	lpg2282	250	lpg2880
	39	lpg2157	*sdeA*	39	lpg0395	92	lpg0701	145	lpg1447	198	lpg2297	251	lpg2882
	40	lpg2156	*sdeB*	40	lpg0399	93	lpg0702	146	lpg1459	199	lpg2303	252	lpg2889
	41	lpg2153	*sdeC*	41	lpg0400	94	lpg0703	147	lpg1463	200	lpg2308	253	lpg2903
	42	lpg2509	*sdeD*	42	lpg0414	95	lpg0725	148	lpg1472	201	lpg2321	254	lpg2905
	43	lpg2154	*sde*	43	lpg0418	96	lpg0738	149	lpg1478	202	lpg2336	255	lpg2933
	44	lpg0376	*sdhA*	44	lpg0426	97	lpg0756	150	lpg1502	203	lpg2338	256	lpg2934
	45	lpg0135	*sdhB*	45	lpg0441	98	lpg0760	151	lpg1504	204	lpg2347	257	lpg2935
	46	lpg2155	*sidJ*	46	lpg0442	99	lpg0781	152	lpg1511	205	lpg2358	258	lpg2937
	47	lpg2508	*sdjA*	47	lpg0443	100	lpg0785	153	lpg1529	206	lpg2487	259	lpg2955
	48	lpg2464	*sidM/drrA*	48	lpg0444	101	lpg0801	154	lpg1530	207	lpg2496	260	lpg2966
	49	lpg0390	*vipA*	49	lpg0445	102	lpg0805	155	lpg1534	208	lpg2594	261	lpg2967
	50	lpg2831	*vipD*	50	lpg0446	103	lpg0811	156	lpg1547	209	lpg2595	262	lpg2980
	51	lpg0103	*vipF*	51	lpg0447	104	lpg0814	157	lpg1548	210	lpg2608	263	lpg2982
	52	lpg2410	*vpdA*	52	lpg0448	105	lpg0816	158	lpg1554	211	lpg2624	264	lpg2983
	53	lpg1227	*vpdB*	53	lpg0449	106	lpg0817	159	lpg1576	212	lpg2625	265	lpg2984

2nd	effectors			non effectors									
	#	lpg	Symbol	#		#							
	1	lpg1950	*ralF*	1	lpg0001	83	lpg0458	165	lpg0954	247	lpg1711	329	lpg2484
	2	lpg2793	*lepA*	2	lpg0004	84	lpg0459	166	lpg0958	248	lpg1712	330	lpg2487
	3	lpg2490	*lepB*	3	lpg0009	85	lpg0460	167	lpg0962	249	lpg1714	331	lpg2496
	4	lpg0940	*lidA*	4	lpg0010	86	lpg0462	168	lpg0977	250	lpg1719	332	lpg2594
	5	lpg2718	*wipA*	5	lpg0052	87	lpg0476	169	lpg1054	251	lpg1721	333	lpg2595
	6	lpg0642	*wipB*	6	lpg0072	88	lpg0479	170	lpg1059	252	lpg1722	334	lpg2608
	7	lpg2298	*ylfA/legC7*	7	lpg0089	89	lpg0481	171	lpg1131	253	lpg1723	335	lpg2609
	8	lpg1884	*ylfB/legC2*	8	lpg0092	90	lpg0486	172	lpg1140	254	lpg1724	336	lpg2617
	9	lpg0284	*ceg10*	9	lpg0093	91	lpg0501	173	lpg1141	255	lpg1726	337	lpg2624
	10	lpg1621	*ceg23*	10	lpg0094	92	lpg0510	174	lpg1179	256	lpg1729	338	lpg2625
	11	lpg2409	*ceg29*	11	lpg0097	93	lpg0513	175	lpg1181	257	lpg1730	339	lpg2627
	12	lpg0898	*ceg18*	12	lpg0099	94	lpg0525	176	lpg1182	258	lpg1731	340	lpg2635
	13	lpg2591	*ceg33*	13	lpg0117	95	lpg0530	177	lpg1193	259	lpg1746	341	lpg2636
	14	lpg0227	*ceg7*	14	lpg0118	96	lpg0531	178	lpg1194	260	lpg1748	342	lpg2646
	15	lpg0012	*cegC1*	15	lpg0125	97	lpg0532	179	lpg1197	261	lpg1750	343	lpg2649
	16	lpg0126	*cegC2*	16	lpg0127	98	lpg0533	180	lpg1200	262	lpg1757	344	lpg2650
	17	lpg1144	*cegC3*	17	lpg0137	99	lpg0534	181	lpg1202	263	lpg1759	345	lpg2654
	18	lpg2200	*cegC4*	18	lpg0139	100	lpg0535	182	lpg1215	264	lpg1766	346	lpg2667
	19	lpg0038	*legA10/ankQ*	19	lpg0141	101	lpg0543	183	lpg1221	265	lpg1767	347	lpg2669
	20	lpg0436	*legA11/ankJ*	20	lpg0157	102	lpg0548	184	lpg1231	266	lpg1770	348	lpg2670

21	lpg1488	*legC5*	21 lpg0194	103 lpg0552	185 lpg1282	267 lpg1771	349 lpg2674
22	lpg1660	*legL3*	22 lpg0218	104 lpg0554	186 lpg1284	268 lpg1777	350 lpg2675
23	lpg1958	*legL5*	23 lpg0250	105 lpg0557	187 lpg1286	269 lpg1778	351 lpg2676
24	lpg2400	*legL7*	24 lpg0252	106 lpg0560	188 lpg1287	270 lpg1789	352 lpg2686
25	lpg1948	*legLC4*	25 lpg0287	107 lpg0577	189 lpg1297	271 lpg1791	353 lpg2687
26	lpg1890	*legLC8*	26 lpg0299	108 lpg0578	190 lpg1304	272 lpg1799	354 lpg2688
27	lpg0621	*sidA*	27 lpg0314	109 lpg0587	191 lpg1306	273 lpg1801	355 lpg2689
28	lpg1642	*sidB*	28 lpg0317	110 lpg0592	192 lpg1307	274 lpg1805	356 lpg2696
29	lpg2511	*sidC*	29 lpg0318	111 lpg0601	193 lpg1319	275 lpg1808	357 lpg2701
30	lpg2465	*sidD*	30 lpg0319	112 lpg0602	194 lpg1334	276 lpg1819	358 lpg2702
31	lpg0234	*sidE*	31 lpg0321	113 lpg0604	195 lpg1341	277 lpg1821	359 lpg2706
32	lpg2584	*sidF*	32 lpg0322	114 lpg0616	196 lpg1362	278 lpg1833	360 lpg2707
33	lpg1355	*sidG*	33 lpg0323	115 lpg0618	197 lpg1364	279 lpg1840	361 lpg2709
34	lpg2829	*sidH*	34 lpg0324	116 lpg0636	198 lpg1369	280 lpg1842	362 lpg2712
35	lpg0275	*sdbA*	35 lpg0326	117 lpg0639	199 lpg1373	281 lpg1853	363 lpg2713
36	lpg2482	*sdbB*	36 lpg0327	118 lpg0640	200 lpg1374	282 lpg1854	364 lpg2714
37	lpg2391	*sdbC*	37 lpg0328	119 lpg0641	201 lpg1383	283 lpg1858	365 lpg2725
38	lpg2510	*sdcA*	38 lpg0329	120 lpg0651	202 lpg1384	284 lpg1859	366 lpg2741
39	lpg2157	*sdeA*	39 lpg0330	121 lpg0659	203 lpg1388	285 lpg1860	367 lpg2742
40	lpg2156	*sdeB*	40 lpg0332	122 lpg0661	204 lpg1389	286 lpg1861	368 lpg2756
41	lpg2153	*sdeC*	41 lpg0334	123 lpg0685	205 lpg1393	287 lpg1872	369 lpg2763
42	lpg2509	*sdeD*	42 lpg0335	124 lpg0688	206 lpg1394	288 lpg1906	370 lpg2764
43	lpg2154	*sde*	43 lpg0336	125 lpg0691	207 lpg1395	289 lpg1911	371 lpg2765
44	lpg0376	*sdhA*	44 lpg0338	126 lpg0694	208 lpg1396	290 lpg1927	372 lpg2768
45	lpg0135	*sdhB*	45 lpg0339	127 lpg0698	209 lpg1397	291 lpg1928	373 lpg2773
46	lpg2155	*sidJ*	46 lpg0341	128 lpg0701	210 lpg1399	292 lpg2004	374 lpg2782
47	lpg2508	*sdjA*	47 lpg0343	129 lpg0702	211 lpg1415	293 lpg2009	375 lpg2794
48	lpg2464	*sidM/drrA*	48 lpg0344	130 lpg0703	212 lpg1417	294 lpg2010	376 lpg2795
49	lpg0390	*vipA*	49 lpg0345	131 lpg0715	213 lpg1420	295 lpg2012	377 lpg2796
50	lpg2831	*vipD*	50 lpg0346	132 lpg0719	214 lpg1421	296 lpg2022	378 lpg2797
51	lpg0103	*vipF*	51 lpg0349	133 lpg0725	215 lpg1425	297 lpg2023	379 lpg2810
52	lpg2410	*vpdA*	52 lpg0351	134 lpg0728	216 lpg1438	298 lpg2024	380 lpg2812
53	lpg1227	*vpdB*	53 lpg0353	135 lpg0738	217 lpg1443	299 lpg2025	381 lpg2822
54	lpg0045	-	54 lpg0354	136 lpg0756	218 lpg1447	300 lpg2028	382 lpg2833
55	lpg0081	-	55 lpg0355	137 lpg0758	219 lpg1459	301 lpg2037	383 lpg2834
56	lpg0294	-	56 lpg0384	138 lpg0760	220 lpg1461	302 lpg2075	384 lpg2836
57	lpg0365	-	57 lpg0388	139 lpg0781	221 lpg1462	303 lpg2189	385 lpg2839
58	lpg0518	-	58 lpg0395	140 lpg0785	222 lpg1463	304 lpg2243	386 lpg2868
59	lpg0634	-	59 lpg0396	141 lpg0801	223 lpg1472	305 lpg2282	387 lpg2872
60	lpg0963	-	60 lpg0399	142 lpg0805	224 lpg1478	306 lpg2284	388 lpg2880
61	lpg1148	-	61 lpg0400	143 lpg0811	225 lpg1502	307 lpg2293	389 lpg2881
62	lpg1158	-	62 lpg0414	144 lpg0814	226 lpg1504	308 lpg2295	390 lpg2882
63	lpg1273	-	63 lpg0416	145 lpg0816	227 lpg1511	309 lpg2297	391 lpg2889
64	lpg1689	-	64 lpg0418	146 lpg0817	228 lpg1528	310 lpg2303	392 lpg2890
65	lpg1717	-	65 lpg0426	147 lpg0818	229 lpg1529	311 lpg2304	393 lpg2903
66	lpg1751	-	66 lpg0433	148 lpg0835	230 lpg1530	312 lpg2307	394 lpg2905
67	lpg2327	-	67 lpg0441	149 lpg0836	231 lpg1534	313 lpg2308	395 lpg2923
68	lpg2407	-	68 lpg0442	150 lpg0839	232 lpg1537	314 lpg2309	396 lpg2933
69	lpg2527	-	69 lpg0443	151 lpg0840	233 lpg1538	315 lpg2314	397 lpg2934
70	lpg2744	-	70 lpg0444	152 lpg0841	234 lpg1541	316 lpg2318	398 lpg2935
71	lpg2830	*legU2/lubX*	71 lpg0445	153 lpg0842	235 lpg1544	317 lpg2321	399 lpg2937
72	lpg0240	*ceg8*	72 lpg0446	154 lpg0847	236 lpg1547	318 lpg2326	400 lpg2955
73	lpg0437	*ceg14*	73 lpg0447	155 lpg0872	237 lpg1548	319 lpg2336	401 lpg2964
74	lpg1426	*vpdC*	74 lpg0448	156 lpg0888	238 lpg1554	320 lpg2338	402 lpg2966
75	lpg1496	*lem10*	75 lpg0449	157 lpg0914	239 lpg1576	321 lpg2347	403 lpg2967
76	lpg1625	*lem12*	76 lpg0450	158 lpg0932	240 lpg1589	322 lpg2353	404 lpg2980
77	lpg1933	*lem15*	77 lpg0451	159 lpg0937	241 lpg1591	323 lpg2357	405 lpg2982
78	lpg2216	*lem20*	78 lpg0452	160 lpg0938	242 lpg1592	324 lpg2358	406 lpg2983
79	lpg2433	*ceg30*	79 lpg0453	161 lpg0942	243 lpg1690	325 lpg2361	407 lpg2984
80	lpg2504	*ceg32*	80 lpg0454	162 lpg0946	244 lpg1696	326 lpg2389	408 lpg2988
81	lpg2523	*lem26*	81 lpg0455	163 lpg0949	245 lpg1704	327 lpg2440	409 lpg2993
82	lpg2826	*ceg34*	82 lpg0456	164 lpg0951	246 lpg1707	328 lpg2475	410 lpg3001

3rd

**#**	**lpg** (effectors)	**Symbol**	**#** (non effectors)				
1	lpg1950	*ralF*	1 lpg0001	135 lpg0533	269 lpg1217	403 lpg1748	537 lpg2593
2	lpg2793	*lepA*	2 lpg0002	136 lpg0534	270 lpg1221	404 lpg1750	538 lpg2594
3	lpg2490	*lepB*	3 lpg0004	137 lpg0535	271 lpg1223	405 lpg1753	539 lpg2595
4	lpg0940	*lidA*	4 lpg0006	138 lpg0543	272 lpg1231	406 lpg1757	540 lpg2597
5	lpg2718	*wipA*	5 lpg0009	139 lpg0548	273 lpg1232	407 lpg1759	541 lpg2608
6	lpg0642	*wipB*	6 lpg0010	140 lpg0552	274 lpg1235	408 lpg1762	542 lpg2609
7	lpg2298	*ylfA/legC7*	7 lpg0022	141 lpg0554	275 lpg1280	409 lpg1764	543 lpg2610
8	lpg1884	*ylfB/legC2*	8 lpg0025	142 lpg0557	276 lpg1282	410 lpg1766	544 lpg2614
9	lpg0284	*ceg10*	9 lpg0052	143 lpg0560	277 lpg1284	411 lpg1767	545 lpg2615
10	lpg1621	*ceg23*	10 lpg0072	144 lpg0561	278 lpg1286	412 lpg1768	546 lpg2616
11	lpg2409	*ceg29*	11 lpg0077	145 lpg0577	279 lpg1287	413 lpg1770	547 lpg2617
12	lpg0898	*ceg18*	12 lpg0079	146 lpg0578	280 lpg1288	414 lpg1771	548 lpg2618
13	lpg2591	*ceg33*	13 lpg0089	147 lpg0586	281 lpg1292	415 lpg1777	549 lpg2624

14 lpg0227	*ceg7*	14 lpg0092	148 lpg0587	282 lpg1293	416 lpg1778	550 lpg2625
15 lpg0012	*cegC1*	15 lpg0093	149 lpg0590	283 lpg1297	417 lpg1782	551 lpg2627
16 lpg0126	*cegC2*	16 lpg0094	150 lpg0592	284 lpg1302	418 lpg1785	552 lpg2631
17 lpg1144	*cegC3*	17 lpg0097	151 lpg0597	285 lpg1304	419 lpg1789	553 lpg2635
18 lpg2200	*cegC4*	18 lpg0099	152 lpg0601	286 lpg1306	420 lpg1791	554 lpg2636
19 lpg0038	*legA10/ankQ*	19 lpg0104	153 lpg0602	287 lpg1307	421 lpg1799	555 lpg2645
20 lpg0436	*legA11/ankJ*	20 lpg0117	154 lpg0604	288 lpg1319	422 lpg1801	556 lpg2646
21 lpg1488	*legC5*	21 lpg0118	155 lpg0607	289 lpg1331	423 lpg1805	557 lpg2649
22 lpg1660	*legL3*	22 lpg0122	156 lpg0612	290 lpg1332	424 lpg1808	558 lpg2650
23 lpg1958	*legL5*	23 lpg0125	157 lpg0616	291 lpg1333	425 lpg1813	559 lpg2653
24 lpg2400	*legL7*	24 lpg0127	158 lpg0618	292 lpg1334	426 lpg1815	560 lpg2654
25 lpg1948	*legLC4*	25 lpg0136	159 lpg0636	293 lpg1341	427 lpg1818	561 lpg2656
26 lpg1890	*legLC8*	26 lpg0137	160 lpg0639	294 lpg1348	428 lpg1819	562 lpg2657
27 lpg0621	*sidA*	27 lpg0138	161 lpg0640	295 lpg1353	429 lpg1821	563 lpg2659
28 lpg1642	*sidB*	28 lpg0139	162 lpg0641	296 lpg1357	430 lpg1825	564 lpg2664
29 lpg2511	*sidC*	29 lpg0141	163 lpg0651	297 lpg1362	431 lpg1829	565 lpg2667
30 lpg2465	*sidD*	30 lpg0157	164 lpg0654	298 lpg1364	432 lpg1833	566 lpg2669
31 lpg0234	*sidE*	31 lpg0166	165 lpg0659	299 lpg1366	433 lpg1835	567 lpg2670
32 lpg2584	*sidF*	32 lpg0194	166 lpg0660	300 lpg1369	434 lpg1839	568 lpg2674
33 lpg1355	*sidG*	33 lpg0218	167 lpg0661	301 lpg1373	435 lpg1840	569 lpg2675
34 lpg2829	*sidH*	34 lpg0230	168 lpg0664	302 lpg1374	436 lpg1842	570 lpg2676
35 lpg0275	*sdbA*	35 lpg0238	169 lpg0682	303 lpg1375	437 lpg1844	571 lpg2686
36 lpg2482	*sdbB*	36 lpg0250	170 lpg0685	304 lpg1376	438 lpg1846	572 lpg2687
37 lpg2391	*sdbC*	37 lpg0252	171 lpg0688	305 lpg1383	439 lpg1853	573 lpg2688
38 lpg2510	*sdcA*	38 lpg0271	172 lpg0691	306 lpg1384	440 lpg1854	574 lpg2689
39 lpg2157	*sdeA*	39 lpg0277	173 lpg0694	307 lpg1388	441 lpg1858	575 lpg2696
40 lpg2156	*sdeB*	40 lpg0286	174 lpg0698	308 lpg1389	442 lpg1859	576 lpg2699
41 lpg2153	*sdeC*	41 lpg0287	175 lpg0701	309 lpg1392	443 lpg1860	577 lpg2701
42 lpg2509	*sdeD*	42 lpg0288	176 lpg0702	310 lpg1393	444 lpg1861	578 lpg2702
43 lpg2154	*sde*	43 lpg0293	177 lpg0703	311 lpg1394	445 lpg1862	579 lpg2706
44 lpg0376	*sdhA*	44 lpg0299	178 lpg0715	312 lpg1395	446 lpg1868	580 lpg2707
45 lpg0135	*sdhB*	45 lpg0314	179 lpg0719	313 lpg1396	447 lpg1869	581 lpg2709
46 lpg2155	*sidJ*	46 lpg0317	180 lpg0725	314 lpg1397	448 lpg1872	582 lpg2710
47 lpg2508	*sdjA*	47 lpg0318	181 lpg0726	315 lpg1399	449 lpg1906	583 lpg2711
48 lpg2464	*sidM/drrA*	48 lpg0319	182 lpg0728	316 lpg1402	450 lpg1911	584 lpg2712
49 lpg0390	*vipA*	49 lpg0320	183 lpg0729	317 lpg1403	451 lpg1916	585 lpg2713
50 lpg2831	*vipD*	50 lpg0321	184 lpg0738	318 lpg1411	452 lpg1919	586 lpg2714
51 lpg0103	*vipF*	51 lpg0322	185 lpg0739	319 lpg1413	453 lpg1922	587 lpg2719
52 lpg2410	*vpdA*	52 lpg0323	186 lpg0745	320 lpg1414	454 lpg1927	588 lpg2725
53 lpg1227	*vpdB*	53 lpg0324	187 lpg0756	321 lpg1415	455 lpg1928	589 lpg2735
54 lpg0045	-	54 lpg0325	188 lpg0758	322 lpg1417	456 lpg1970	590 lpg2741
55 lpg0081	-	55 lpg0326	189 lpg0760	323 lpg1418	457 lpg2000	591 lpg2742
56 lpg0294	-	56 lpg0327	190 lpg0781	324 lpg1420	458 lpg2001	592 lpg2743
57 lpg0365	-	57 lpg0328	191 lpg0785	325 lpg1421	459 lpg2004	593 lpg2754
58 lpg0518	-	58 lpg0329	192 lpg0790	326 lpg1423	460 lpg2006	594 lpg2756
59 lpg0634	-	59 lpg0330	193 lpg0800	327 lpg1425	461 lpg2009	595 lpg2763
60 lpg0963	-	60 lpg0332	194 lpg0801	328 lpg1434	462 lpg2010	596 lpg2764
61 lpg1148	-	61 lpg0334	195 lpg0805	329 lpg1438	463 lpg2011	597 lpg2765
62 lpg1158	-	62 lpg0335	196 lpg0807	330 lpg1439	464 lpg2012	598 lpg2768
63 lpg1273	-	63 lpg0336	197 lpg0811	331 lpg1441	465 lpg2013	599 lpg2770
64 lpg1689	-	64 lpg0338	198 lpg0814	332 lpg1443	466 lpg2014	600 lpg2772
65 lpg1717	-	65 lpg0339	199 lpg0816	333 lpg1447	467 lpg2022	601 lpg2773
66 lpg1751	-	66 lpg0340	200 lpg0817	334 lpg1457	468 lpg2023	602 lpg2782
67 lpg2327	-	67 lpg0341	201 lpg0818	335 lpg1459	469 lpg2024	603 lpg2784
68 lpg2407	-	68 lpg0343	202 lpg0826	336 lpg1461	470 lpg2025	604 lpg2785
69 lpg2527	-	69 lpg0344	203 lpg0834	337 lpg1462	471 lpg2026	605 lpg2788
70 lpg2744	-	70 lpg0345	204 lpg0835	338 lpg1463	472 lpg2028	606 lpg2792
71 lpg2830	*legU2/lubX*	71 lpg0346	205 lpg0836	339 lpg1467	473 lpg2033	607 lpg2794
72 lpg0240	*ceg8*	72 lpg0348	206 lpg0839	340 lpg1472	474 lpg2037	608 lpg2795
73 lpg0437	*ceg14*	73 lpg0349	207 lpg0840	341 lpg1478	475 lpg2043	609 lpg2796
74 lpg1426	*vpdC*	74 lpg0351	208 lpg0841	342 lpg1480	476 lpg2075	610 lpg2797
75 lpg1496	*lem10*	75 lpg0352	209 lpg0842	343 lpg1486	477 lpg2099	611 lpg2802
76 lpg1625	*lem12*	76 lpg0353	210 lpg0847	344 lpg1502	478 lpg2111	612 lpg2805
77 lpg1933	*lem15*	77 lpg0354	211 lpg0848	345 lpg1503	479 lpg2122	613 lpg2809
78 lpg2216	*lem20*	78 lpg0355	212 lpg0855	346 lpg1504	480 lpg2132	614 lpg2810
79 lpg2433	*ceg30*	79 lpg0356	213 lpg0856	347 lpg1509	481 lpg2189	615 lpg2811
80 lpg2504	*ceg32*	80 lpg0357	214 lpg0858	348 lpg1511	482 lpg2191	616 lpg2812
81 lpg2523	*lem26*	81 lpg0366	215 lpg0860	349 lpg1522	483 lpg2222	617 lpg2816
82 lpg2826	*ceg34*	82 lpg0384	216 lpg0861	350 lpg1528	484 lpg2243	618 lpg2817
83 lpg1836	*ceg25*	83 lpg0387	217 lpg0862	351 lpg1529	485 lpg2247	619 lpg2822
84 lpg1960	*lirA*	84 lpg0388	218 lpg0867	352 lpg1530	486 lpg2263	620 lpg2823
85 lpg1962	*lirB*	85 lpg0395	219 lpg0872	353 lpg1534	487 lpg2282	621 lpg2824
86 lpg1963	*lirC*	86 lpg0396	220 lpg0874	354 lpg1537	488 lpg2284	622 lpg2833
87 lpg1964	*lirD*	87 lpg0397	221 lpg0876	355 lpg1538	489 lpg2287	623 lpg2834
88 lpg1965	*lirE*	88 lpg0399	222 lpg0887	356 lpg1539	490 lpg2293	624 lpg2836
89 lpg1966	*lirF*	89 lpg0400	223 lpg0888	357 lpg1541	491 lpg2295	625 lpg2839
90 lpg0403	*legA7/ankG/ankZ*	90 lpg0414	224 lpg0899	358 lpg1544	492 lpg2297	626 lpg2868
91 lpg0402	*legA9/ceg12/ankY*	91 lpg0416	225 lpg0903	359 lpg1547	493 lpg2299	627 lpg2869
92 lpg2300	*legA3/ankH/ankW*	92 lpg0418	226 lpg0914	360 lpg1548	494 lpg2302	628 lpg2871

93 lpg0695	*legA8/ankN/ankX*	93 lpg0420	227 lpg0916	361 lpg1554	495 lpg2303	629 lpg2872
94 lpg0483	*legA12/ankC*	94 lpg0426	228 lpg0932	362 lpg1568	496 lpg2304	630 lpg2873
95 lpg2456	*legA15/ankD*	95 lpg0433	229 lpg0933	363 lpg1571	497 lpg2307	631 lpg2875
96 lpg2215	*legA2*	96 lpg0441	230 lpg0936	364 lpg1574	498 lpg2308	632 lpg2880
97 lpg2322	*legA5/ankK*	97 lpg0442	231 lpg0937	365 lpg1576	499 lpg2309	633 lpg2881
98 lpg1718	*legAS4/ankI*	98 lpg0443	232 lpg0938	366 lpg1583	500 lpg2314	634 lpg2882
99 lpg2144	*legAU13/ceg27/ankB*	99 lpg0444	233 lpg0942	367 lpg1589	501 lpg2318	635 lpg2883
100 lpg1701	*legC3*	100 lpg0445	234 lpg0946	368 lpg1591	502 lpg2319	636 lpg2887
101 lpg1953	*legC4*	101 lpg0446	235 lpg0949	369 lpg1592	503 lpg2321	637 lpg2889
102 lpg1588	*legC6*	102 lpg0447	236 lpg0951	370 lpg1595	504 lpg2326	638 lpg2890
103 lpg2862	*legC8*	103 lpg0448	237 lpg0952	371 lpg1609	505 lpg2335	639 lpg2903
104 lpg1976	*legG1*	104 lpg0449	238 lpg0954	372 lpg1624	506 lpg2336	640 lpg2905
105 lpg0276	*legG2*	105 lpg0450	239 lpg0958	373 lpg1626	507 lpg2337	641 lpg2923
106 lpg2137	*legK2*	106 lpg0451	240 lpg0962	374 lpg1634	508 lpg2338	642 lpg2926
107 lpg2556	*legK3*	107 lpg0452	241 lpg0977	375 lpg1672	509 lpg2340	643 lpg2928
108 lpg0945	*legL1*	108 lpg0453	242 lpg1020	376 lpg1674	510 lpg2345	644 lpg2929
109 lpg1602	*legL2*	109 lpg0454	243 lpg1024	377 lpg1677	511 lpg2347	645 lpg2933
110 lpg2999	*legP*	110 lpg0455	244 lpg1048	378 lpg1682	512 lpg2353	646 lpg2934
111 lpg0171	*legU1*	111 lpg0456	245 lpg1054	379 lpg1690	513 lpg2354	647 lpg2935
112 lpg2452	*legA14*	112 lpg0458	246 lpg1059	380 lpg1696	514 lpg2357	648 lpg2936
113 lpg2176	*legS2*	113 lpg0459	247 lpg1096	381 lpg1699	515 lpg2358	649 lpg2937
114 lpg0090	*lem1*	114 lpg0460	248 lpg1105	382 lpg1703	516 lpg2360	650 lpg2943
115 lpg0191	*ceg5*	115 lpg0462	249 lpg1131	383 lpg1704	517 lpg2361	651 lpg2945
116 lpg0285	*lem2*	116 lpg0469	250 lpg1138	384 lpg1707	518 lpg2381	652 lpg2947
117 lpg0519	*ceg17*	117 lpg0476	251 lpg1139	385 lpg1708	519 lpg2384	653 lpg2955
118 lpg0696	*lem3*	118 lpg0477	252 lpg1140	386 lpg1711	520 lpg2389	654 lpg2963
119 lpg1101	*lem4*	119 lpg0479	253 lpg1141	387 lpg1712	521 lpg2440	655 lpg2964
120 lpg1110	*lem5*	120 lpg0481	254 lpg1174	388 lpg1713	522 lpg2454	656 lpg2965
121 lpg1120	*lem6*	121 lpg0484	255 lpg1179	389 lpg1714	523 lpg2471	657 lpg2966
122 lpg1145	*lem7*	122 lpg0486	256 lpg1180	390 lpg1719	524 lpg2472	658 lpg2967
123 lpg1290	*lem8*	123 lpg0493	257 lpg1181	391 lpg1720	525 lpg2475	659 lpg2971
124 lpg1491	*lem9*	124 lpg0495	258 lpg1182	392 lpg1721	526 lpg2476	660 lpg2974
125 lpg1598	*lem11*	125 lpg0501	259 lpg1193	393 lpg1722	527 lpg2478	661 lpg2980
126 lpg1702	*lem13*	126 lpg0503	260 lpg1194	394 lpg1723	528 lpg2484	662 lpg2982
127 lpg1851	*lem14*	127 lpg0505	261 lpg1195	395 lpg1724	529 lpg2487	663 lpg2983
128 lpg1949	*lem17*	128 lpg0510	262 lpg1197	396 lpg1726	530 lpg2488	664 lpg2984
129 lpg1969	*lem18*	129 lpg0511	263 lpg1199	397 lpg1729	531 lpg2496	665 lpg2986
130 lpg2248	*lem21*	130 lpg0513	264 lpg1200	398 lpg1730	532 lpg2517	666 lpg2988
131 lpg2328	*lem22*	131 lpg0525	265 lpg1202	399 lpg1731	533 lpg2530	667 lpg2993
132 lpg2411	*lem24*	132 lpg0530	266 lpg1203	400 lpg1744	534 lpg2534	668 lpg2996
133 lpg2422	*lem25*	133 lpg0531	267 lpg1204	401 lpg1746	535 lpg2553	669 lpg3001
134 lpg2603	*lem28*	134 lpg0532	268 lpg1215	402 lpg1747	536 lpg2573	670 lpg3002

- Genes we validated in previous phases
