# Supplementary material for: Genome-Scale Identification of Legionella pneumophila Effectors Using a Machine Learning Approach
Source: PLoS Pathog. 2009 Jul 10;5(7):e1000508. doi: 10.1371/journal.ppat.1000508 (PMC2701608; doi:10.1371/journal.ppat.1000508)
Supplement: Table S2 — (0.10 MB PDF) [file ppat.1000508.s003.pdf]

1st phase learning

Best performing classifier (AUC: 0.9944): Voting of the following classifiers*:
• Naïve Bayes with features**: 2, 7, 37, 38, 41, 42, 44
• Bayesian network using 'repeated hill climbing' search algorithm with maximum 2 parent nodes; features: 2, 7, 12, 36, 37, 38, 41, 42, 44
• SVM (SMO) using logistic models and standardization of training set; features: 2, 3, 12, 19, 21, 23, 24, 27, 30, 31, 34, 38, 40, 41, 42, 43, 44
• Multilayer perceptron with decaying learning rate; learning rate initialized to 1; 500 training epochs;
Features:1, 2, 7, 12, 19, 23, 24, 25, 28, 30, 31, 33, 34, 36, 37, 38, 40, 41, 42, 43, 44.

* - Where not explicitly noted otherwise, WEKA default parameters were used

** - Feature table appears hereafter

1st phase summary: 11 / 11 / 12 (validated / cloned and expressed / tested)

Rank	ORF	Symbol	Classification score
**1**	**lpg2523**		0.99
**2**	**lpg1484**	**bad clone**	0.99
**3**	**lpg1496**		0.99
**4**	**lpg2504**	***ceg32***	0.99
**5**	**lpg1933**		0.99
**6**	**lpg2452**	***legA14 \ ceg31***	0.99
**7**	**lpg2392**	***legL6***	0.99
**8**	**lpg1426**	***vpdC***	0.99
**9**	**lpg1963**	***lirC***	0.99
**10**	**lpg0437**	***ceg14***	0.99
**11**	**lpg2433**	***ceg30***	0.99
**12**	**lpg2216**		0.98
**13**	**lpg1625**		0.98
**14**	**lpg2826**	***ceg34***	0.98
**15**	**lpg0240**	***ceg8***	0.97
16	lpg2144	*ceg27\legAU13*	0.97
17	lpg0285		0.97
18	lpg1931		0.96
19	lpg2813	*vipE*	0.96
20	lpg1317		0.96
21	lpg0945	*legL1*	0.96
22	lpg1666	*ceg24*	0.96
23	lpg1290		0.95
24	lpg1947		0.95
25	lpg1121	*ceg19*	0.95
26	lpg0236		0.95
27	lpg1120		0.95
28	lpg1483	*legK1*	0.95
29	lpg0230		0.95

- genes tested
- highly probable effectors according to previous studies, which were hence not tested
- prediction not tested

2nd phase learning

Best performing classifier*: Baysian network (AUC: 0.9947);
Using K2 search algorithm
Features selected**: 1, 2, 4, 36, 38, 39, 41, 43 ,44

* - Where not explicitly noted otherwise, WEKA default parameters were used
** - Feature table appears hereafter

2nd phase summary: 21 / 24 / 25 (validated / cloned and expressed / tested)

Rank	ORF	Symbol	Classification score
**1**	**lpg0090**		1
**2**	**lpg0191**	***ceg5***	1
**3**	**lpg0285**		1
**4**	**lpg0502**	**not traslocated**	1
**5**	**lpg0519**	***ceg17***	1
**6**	**lpg0696**		1
**7**	**lpg0744**		1
**8**	**lpg0945**	***legL1***	1
**9**	**lpg1101**		1
**10**	**lpg1120**		1
**11**	**lpg1149**		1
**12**	**lpg1290**		1
**13**	**lpg1368**	***lgt1***	1
**14**	**lpg1455**		1
**15**	**lpg1491**		1
**16**	**lpg1588**	***legC6***	1
**17**	**lpg1598**		1
**18**	**lpg1701**	***legC3***	1
**19**	**lpg1851**		1
**20**	**lpg1963**	***lirC***	1
**21**	**lpg1969**		1
**22**	**lpg2129**		1
**23**	**lpg2206**	**bad clone**	1
**24**	**lpg2239**		1
**25**	**lpg2248**		1
**26**	**lpg2328**		1
**27**	**lpg2392**	***legL6***	1
**28**	**lpg2422**		1
**29**	**lpg2452**	***legA14\ceg31***	1
**30**	**lpg2546**		1
**31**	**lpg2603**		1
**32**	**lpg2862**	***legC8***	1
**33**	**lpg0196**	**bad clone**	1
**34**	**lpg0401**	***ceg11***	1
**35**	**lpg1110**		1
**36**	**lpg1317**		1
**37**	**lpg1483**	***legK1***	1
**38**	**lpg1602**	***legL2***	1
**39**	**lpg1702**		1
**40**	**lpg2507**	**not traslocated**	1
**41**	**lpg2762**		1
**42**	**lpg1145**		1
**43**	**lpg1489**		1
**44**	**lpg1716**		1
**45**	**lpg1949**		1
**46**	**lpg1959**		1
**47**	**lpg2199**		1
**48**	**lpg2411**		1
**49**	**lpg2717**		1
**50**	**lpg2832**		1
51	lpg0035	*ceg1*	1
52	lpg0054		1
53	lpg0973		1
54	lpg1067		1
55	lpg1109		1
56	lpg1171	*ceg21*	1

- genes tested
- highly probable effectors according to previous studies, which were hence not tested
- prediction not tested

57	lpg1666	*ceg24*	1
58	lpg1683		1
59	lpg1684		1
60	lpg2109		1
61	lpg2144	*ceg27 \ legAU13*	1
62	lpg2444		1
63	lpg1111		1
64	lpg2804		1
65	lpg2813	*vipE*	1
66	lpg0044		1
67	lpg1718	*legAS4*	1
68	lpg1947		1
69	lpg0080	*ceg3*	0.99
70	lpg0276	*legG2*	0.99
71	lpg1354		0.99
72	lpg1752		0.99
73	lpg2215	*legA2*	0.99
74	lpg2406		0.99
75	lpg2745		0.99
76	lpg0364		0.99
77	lpg0438		0.99
78	lpg0082		0.99
79	lpg0440	*ceg16*	0.99
80	lpg0716		0.99
81	lpg0722		0.99
82	lpg0763		0.99
83	lpg1493		0.99
84	lpg1527		0.99
85	lpg1931	*ceg26*	0.99
86	lpg0086		0.99
87	lpg0195		0.99
88	lpg0236		0.99
89	lpg0246	*ceg9*	0.99
90	lpg0407		0.99
91	lpg1121	*ceg19*	0.99
92	lpg2806		0.99
93	lpg2952	*ceg35*	0.99
94	lpg2108		0.98
95	lpg1183		0.98
96	lpg2166		0.98
97	lpg0110		0.98
98	lpg0148		0.98
99	lpg0209		0.98
100	lpg0405		0.98
101	lpg0563		0.98
102	lpg0733		0.98
103	lpg0767		0.98
104	lpg0774		0.98
105	lpg0788		0.98
106	lpg0796		0.98
107	lpg0926		0.98
108	lpg1152		0.98
109	lpg1268		0.98
110	lpg1481		0.98
111	lpg1563		0.98
112	lpg1639		0.98
113	lpg1738		0.98
114	lpg1798		0.98
115	lpg1964		0.98
116	lpg2072		0.98
117	lpg2073		0.98
118	lpg2148		0.98
119	lpg2283		0.98
120	lpg2424		0.98
121	lpg2519		0.98
122	lpg2761		0.98
123	lpg2916		0.98
124	lpg1274		0.98
125	lpg1891		0.98

126	lpg1934	0.98
127	lpg0039	0.97
128	lpg0046	0.97
129	lpg0375	0.97
130	lpg1495	0.97
131	lpg1932	0.97
132	lpg2505	0.97
133	lpg0941	0.96
134	lpg0391	0.96
135	lpg0622	0.96
136	lpg2719	0.96

3rd phase learning

Best performing classifier*: Baysian network (AUC: 0.996);
Using K2 search algorithm with maximum two parent nodes
Features selected**: 1, 2, 4, 5, 7, 20, 23, 24, 31, 35, 36, 38, 41, 43, 44

* - Where not explicitly noted otherwise, WEKA default parameters were used
** - Feature table appears hereafter

3rd overall: 8 / 8 / 8 (validated / cloned and expressed / tested)

Rank	ORF	Symbol	Classification score	Genes shown to translocate in Heidtman et al. 2009	Genes shown to translocate in Shin et al. 2008
**1**	**lpg0080**	***ceg3***	**1**		
**2**	**lpg0096**	***ceg4***	**1**		
**3**	**lpg1947**		**1**		
**4**	**lpg2166**		**1**		
**5**	**lpg2406**		**1**		
**6**	**lpg2529**		**1**		
**7**	**lpg2804**		**1**		
**8**	**lpg1121**	***ceg19***	**1**	X	
11	lpg0035	*ceg1*	1		
12	lpg0172		1		
13	lpg0236		1		
14	lpg0401	*ceg11*	1		
15	lpg0440	*ceg16*	1		
16	lpg1109		1		
9	lpg1166		1		
17	lpg1171	*ceg21*	1		
18	lpg1354		1		
19	lpg1368	*lgt1*	1		
20	lpg1455		1		
21	lpg1483	*legK1*	1		X
22	lpg1666	*ceg24*	1		
23	lpg1924		1		
10	lpg1931	*ceg26*	1		
24	lpg1952		1		
25	lpg1957		1		
26	lpg1959		1		
27	lpg1961		1		
28	lpg1975		1		
29	lpg1979		1		
30	lpg1986		1		
31	lpg2143		1		
32	lpg2147		1		
33	lpg2148		1		
34	lpg2159		1		
35	lpg2199		1		
36	lpg2224		1		
37	lpg2244		1		
38	lpg2311	*ceg28*	1		
39	lpg2392	*legL6*	1		
40	lpg2408		1		
41	lpg2424		1		
42	lpg2451		1		
43	lpg2505		1		
44	lpg2519		1		
45	lpg2525		1		
46	lpg2546		1		
47	lpg2745		1		
48	lpg2762		1		
49	lpg2813	*vipE*	1		
50	lpg2976		1		
51	lpg0921		1		
52	lpg1067		1		
53	lpg1111		1		
54	lpg1124		1		
55	lpg1137	*ceg20*	1		
56	lpg1149		1		

-genes tested (green)
-prediction not tested (yellow)

57	lpg1581		1	
58	lpg1683		1	
59	lpg1684		1	
60	lpg1909		1	
61	lpg1951		1	
62	lpg1968		1	
63	lpg1972		1	
64	lpg1980		1	
65	lpg1990		1	
66	lpg2149		1	
67	lpg2150		1	
68	lpg2170		1	
69	lpg2394		1	
70	lpg2395		1	
71	lpg2403		1	
72	lpg2414		1	
73	lpg2425		1	
74	lpg2453		1	
75	lpg2461		1	
76	lpg2498		1	
77	lpg2520		1	
78	lpg2832		1	
79	lpg0059	*ceg2*	1	
80	lpg0062		1	
81	lpg0159		1	
82	lpg0210		1	
83	lpg0246	*ceg9*	1	**X**
84	lpg0796		1	
85	lpg1151		1	
86	lpg1238		1	
87	lpg1263		1	
88	lpg1268		1	
89	lpg1317		1	
90	lpg1436		1	
91	lpg1489		1	
92	lpg1505		1	
93	lpg1639		1	
94	lpg1654		1	
95	lpg1738		1	
96	lpg1925		1	
97	lpg1982		1	
98	lpg2109		1	
99	lpg2129		1	
100	lpg2283		1	
101	lpg2339		1	
102	lpg2344		1	
103	lpg2416	*legA1*	1	
104	lpg2444		1	
105	lpg2724		1	
106	lpg2759		1	
107	lpg0098		1	
108	lpg0693		1	
109	lpg1930		1	
110	lpg2160		1	
111	lpg2413		1	
112	lpg2518		1	
113	lpg0082		1	
114	lpg0716		1	
115	lpg0926		1	
116	lpg0941		1	
117	lpg0968		1	
118	lpg1132		1	
119	lpg1407		1	
120	lpg1983		1	
121	lpg2443		1	
122	lpg2522		1	
123	lpg2555		1	
124	lpg2761		1	
125	lpg3000		1	
126	lpg0039		1	
127	lpg0046		1	
128	lpg0054		1	

129	lpg0189		1	
130	lpg0190		1	
131	lpg0209		1	
132	lpg0247		1	
133	lpg0405		1	
134	lpg1495		1	
135	lpg1978	*setA*	1	**X**
136	lpg2375		1	
137	lpg2542		1	
138	lpg0086		0.99	
139	lpg0407		0.99	
140	lpg0969		0.99	
141	lpg0973		0.99	
142	lpg1954		0.99	
143	lpg2455		0.99	
144	lpg0192		0.99	
145	lpg1102		0.99	
146	lpg1752		0.99	
147	lpg2131	*legA6*	0.99	
148	lpg2151		0.99	
149	lpg2952	*ceg35*	0.99	
150	lpg0030		0.99	
151	lpg0110		0.99	
152	lpg0563		0.99	
153	lpg0733		0.99	
154	lpg0767		0.99	
155	lpg0770		0.99	
156	lpg0771		0.99	
157	lpg0788		0.99	
158	lpg0871		0.99	
159	lpg0974		0.99	
160	lpg1106		0.99	
161	lpg1152		0.99	
162	lpg1318		0.99	
163	lpg1465		0.99	
164	lpg1481		0.99	
165	lpg1551		0.99	
166	lpg2050		0.99	
167	lpg2073		0.99	
168	lpg2239		0.99	
169	lpg2271		0.99	
170	lpg2372		0.99	
171	lpg2502		0.99	
172	lpg2571		0.99	
173	lpg2682		0.99	
174	lpg2162		0.99	
175	lpg2404		0.99	
176	lpg2806		0.99	
177	lpg0148		0.99	
178	lpg0258		0.99	
179	lpg1154		0.99	
180	lpg1233		0.99	
181	lpg1578		0.99	
182	lpg2171		0.99	
183	lpg2717		0.99	
184	lpg1688		0.99	
185	lpg1973		0.99	
186	lpg2165		0.99	
187	lpg2312		0.99	
188	lpg2437		0.99	
189	lpg2445		0.99	
190	lpg0091		0.99	
191	lpg0765		0.99	
192	lpg1798		0.99	
193	lpg1817		0.99	
194	lpg0364		0.99	
195	lpg0375		0.99	
196	lpg1493		0.99	
197	lpg0391		0.99	
198	lpg1887		0.99	
199	lpg1129		0.98	
200	lpg1289		0.98	

201	lpg0878	0.98
202	lpg1309	0.98
203	lpg1310	0.98
204	lpg1527	0.98
205	lpg2446	0.98
206	lpg0196	0.98
207	lpg0042	0.98
208	lpg0181	0.98
209	lpg0393	0.98
210	lpg0901	0.98
211	lpg1237	0.98
212	lpg1261	0.98
213	lpg1265	0.98
214	lpg1270	0.98
215	lpg1670	0.98
216	lpg1754	0.98
217	lpg1881	0.98
218	lpg1895	0.98
219	lpg1907	0.98
220	lpg2223	0.98
221	lpg2257	0.98
222	lpg2382	0.98
223	lpg2538	0.98
224	lpg2539	0.98
225	lpg2541	0.98
226	lpg2572	0.98
227	lpg2803	0.98
228	lpg2874	0.98
229	lpg0111	0.98
230	lpg0201	0.98
231	lpg1995	0.98
232	lpg0722	0.98
233	lpg0763	0.98
234	lpg0130	0.98
235	lpg0160	0.98
236	lpg1716	0.98
237	lpg2220	0.98
238	lpg2552	0.98
239	lpg2879	0.98
240	lpg1267	0.97
241	lpg0044	0.97
242	lpg0268	0.97
243	lpg1932	0.97
244	lpg1991	0.97
245	lpg2385	0.97
246	lpg0182	0.97
247	lpg0229	0.97
248	lpg0480	0.97
249	lpg0509	0.97
250	lpg1091	0.97
251	lpg1298	0.97
252	lpg1429	0.97
253	lpg1431	0.97
254	lpg1593	0.97
255	lpg1668	0.97
256	lpg1681	0.97
257	lpg1687	0.97
258	lpg1876	0.97
259	lpg1902	0.97
260	lpg1937	0.97
261	lpg2124	0.97
262	lpg2195	0.97
263	lpg2412	0.97
264	lpg2497	0.97
265	lpg2543	0.97
266	lpg2561	0.97
267	lpg2563	0.97
268	lpg2569	0.97
269	lpg2846	0.97
270	lpg1563	0.97
271	lpg1803	0.97
272	lpg2371	0.97

273	lpg0431	0.96
274	lpg0766	0.96
275	lpg2351	0.96
276	lpg2386	0.96
277	lpg2526	0.96
278	lpg2435	0.96
279	lpg0112	0.96
280	lpg1492	0.96
281	lpg0920	0.96
282	lpg1133	0.96
283	lpg1622	0.96
284	lpg1981	0.96
285	lpg1099	0.96
286	lpg1498	0.96
287	lpg1644	0.96
288	lpg0195	0.95
289	lpg1967	0.95

Feature index

No.	Feature
1	Blast-P bit score to most similar effector
2	Number of effectors with E-value < 0.01
3	Distance to most proximate effector (in ORFs)
4	Number of effectors 1 ORF away
5	Number of effectors 2 ORFs away
6	Number of effectors 3 ORFs away
7	Number of effectors 4 ORFs away
8	Number of effectors 5 ORFs away
9	Number of effectors 6 ORFs away
10	Number of effectors 7 ORFs away
11	Number of effectors 8 ORFs away
12	Number of effectors 9 ORFs away
13	Number of effectors 10 ORFs away
14	Number of effectors 11 ORFs away
15	Number of effectors 12 ORFs away
16	Number of effectors 13 ORFs away
17	Number of effectors 14 ORFs away
18	Number of effectors 15 ORFs away
19	Number of effectors 16 ORFs away
20	Number of effectors 17 ORFs away
21	Number of effectors 18 ORFs away
22	Number of effectors 19 ORFs away
23	Number of effectors 20 ORFs away
24	Number of effectors 21 ORFs away
25	Number of effectors 22 ORFs away
26	Number of effectors 23 ORFs away
27	Number of effectors 24 ORFs away
28	Number of effectors 25 ORFs away
29	Number of effectors 26 ORFs away
30	Number of effectors 27 ORFs away
31	Number of effectors 28 ORFs away
32	Number of effectors 29 ORFs away
33	Number of effectors 30 ORFs away
34	Highest similarity score to a protein from the humanGnomon predicted proteins
35	Highest similarity score to a protein from the human proteome
36	Highest similarity score to a protein from the *Dictyostelium discoideum* proteome
37	Highest similarity score to a protein from the *Tetrahymena thermophila* proteome
38	PSSM similarity score to PmrA regulatory element
39	Signal score based on tiny, polar, and charged amino-acids
40	Signal based on reported motif from *Helicobacter pylori* effectors
41	G+C content
42	PSSM similarity score to CpxR regulatory element
43	Number of homologs in Metazoa
44	Number of homologs in Bacteria
