# Supplementary material for: Genome-Scale Identification of Legionella pneumophila Effectors Using a Machine Learning Approach
Source: PLoS Pathog. 2009 Jul 10;5(7):e1000508. doi: 10.1371/journal.ppat.1000508 (PMC2701608; doi:10.1371/journal.ppat.1000508)
Supplement: Table S3 — (0.05 MB PDF) [file ppat.1000508.s004.pdf]

Predicted effectors according to the final learning phase

Rank	ORF	Symbol	Classification score
1	lpg0035	*ceg1*	1
2	lpg0172		1
3	lpg0236		1
4	lpg0401	*ceg11*	1
5	lpg0440	*ceg16*	1
6	lpg1109		1
7	lpg1166		1
8	lpg1171	*ceg21*	1
9	lpg1354		1
10	lpg1368	*lgt1*	1
11	lpg1455		1
12	lpg1666	*ceg24*	1
13	lpg1924		1
14	lpg1931	*ceg26*	1
15	lpg1952		1
16	lpg1957		1
17	lpg1959		1
18	lpg1961		1
19	lpg1975		1
20	lpg1979		1
21	lpg1986		1
22	lpg2143		1
23	lpg2147		1
24	lpg2148		1
25	lpg2159		1
26	lpg2199		1
27	lpg2224		1
28	lpg2244		1
29	lpg2311	*ceg28*	1
30	lpg2392	*legL6*	1
31	lpg2408		1
32	lpg2424		1
33	lpg2451		1
34	lpg2505		1
35	lpg2519		1
36	lpg2525		1
37	lpg2546		1
38	lpg2745		1
39	lpg2762		1
40	lpg2813	*vipE*	1
41	lpg2976		1
42	lpg0921		1
43	lpg1067		1
44	lpg1111		1
45	lpg1124		1
46	lpg1137	*ceg20*	1
47	lpg1149		1
48	lpg1581		1
49	lpg1683		1
50	lpg1684		1
51	lpg1909		1
52	lpg1951		1
53	lpg1968		1

54	lpg1972		1
55	lpg1980		1
56	lpg1990		1
57	lpg2149		1
58	lpg2150		1
59	lpg2170		1
60	lpg2394		1
61	lpg2395		1
62	lpg2403		1
63	lpg2414		1
64	lpg2425		1
65	lpg2453		1
66	lpg2461		1
67	lpg2498		1
68	lpg2520		1
69	lpg2832		1
70	lpg0059	*ceg2*	1
71	lpg0062		1
72	lpg0159		1
73	lpg0210		1
74	lpg0796		1
75	lpg1151		1
76	lpg1238		1
77	lpg1263		1
78	lpg1268		1
79	lpg1317		1
80	lpg1436		1
81	lpg1489		1
82	lpg1505		1
83	lpg1639		1
84	lpg1654		1
85	lpg1738		1
86	lpg1925		1
87	lpg1982		1
88	lpg2109		1
89	lpg2129		1
90	lpg2283		1
91	lpg2339		1
92	lpg2344		1
93	lpg2416	*legA1*	1
94	lpg2444		1
95	lpg2724		1
96	lpg2759		1
97	lpg0098		1
98	lpg0693		1
99	lpg1930		1
100	lpg2160		1
101	lpg2413		1
102	lpg2518		1
103	lpg0082		1
104	lpg0716		1
105	lpg0926		1
106	lpg0941		1
107	lpg0968		1
108	lpg1132		1
109	lpg1407		1

110	lpg1983	1
111	lpg2443	1
112	lpg2522	1
113	lpg2555	1
114	lpg2761	1
115	lpg3000	1
116	lpg0039	1
117	lpg0046	1
118	lpg0054	1
119	lpg0189	1
120	lpg0190	1
121	lpg0209	1
122	lpg0247	1
123	lpg0405	1
124	lpg1495	1
125	lpg2375	1
126	lpg2542	1
