# Supplementary material for: Genome-Scale Identification of Legionella pneumophila Effectors Using a Machine Learning Approach
Source: PLoS Pathog. 2009 Jul 10;5(7):e1000508. doi: 10.1371/journal.ppat.1000508 (PMC2701608; doi:10.1371/journal.ppat.1000508)
Supplement: Table S4 — (0.05 MB PDF) [file ppat.1000508.s005.pdf]

C-terminal residues that manifested significant enrichment or depletion

Single positions

Residue	Position	*p-value**
E	12	6.54E-11
E	1	1.54E-06
E	11	2.30E-06
S	5	3.54E-05
E	9	1.20E-04
T	5	7.30E-04
E	13	9.28E-04
S	7	7.60E-03
E	4	9.71E-03
D	14	0.01
D	1	0.01
E	15	0.02
S	6	0.02
D	13	0.03
T	6	0.03
P	6	0.04
D	2	0.04

Sliding window (3aa) on grouped amino-acids

Residue	From position	To position	*p-value**
[ED]	1	3	0
[ST]	5	7	1.10E-13
[ED]	11	13	4.23E-13
[ED]	12	14	5.01E-13
[ST]	4	6	4.26E-12
[ED]	14	16	6.26E-11
[ED]	13	15	1.45E-10
[ED]	2	4	3.09E-09
[ST]	6	8	5.60E-09
[ED]	10	12	5.81E-09
[ST]	7	9	5.97E-07
[ILVF]	9	11	1.58E-06
[ED]	9	11	3.08E-06
[ED]	15	17	3.08E-06
[ED]	3	5	4.49E-06
[ST]	3	5	5.54E-06
[ILVF]	8	10	4.32E-04
[ST]	8	10	5.79E-04
[ST]	9	11	8.01E-04
[ILVF]	20	22	2.01E-03
[RKH]	11	13	2.38E-03
[ILVF]	1	3	2.76E-03
[ILVF]	10	12	3.65E-03
[ILVF]	19	21	6.10E-03
[ED]	8	10	6.27E-03
[NQ]	12	14	7.39E-03
[ED]	4	6	9.26E-03
[ED]	16	18	3.37E-02
[NQ]	13	15	4.35E-02

* After Bonferroni correction
