# Supplementary material for: Genome-Scale Identification of Legionella pneumophila Effectors Using a Machine Learning Approach
Source: PLoS Pathog. 2009 Jul 10;5(7):e1000508. doi: 10.1371/journal.ppat.1000508 (PMC2701608; doi:10.1371/journal.ppat.1000508)
Supplement: Table S5 — (0.03 MB PDF) [file ppat.1000508.s006.pdf]

PmrA PSSM

Position	A	T	C	G
**1**	0.23	0.08	0.62	0.08
**2**	0.08	0.85	0.04	0.04
**3**	0.04	0.88	0.04	0.04
**4**	0.88	0.04	0.04	0.04
**5**	0.88	0.04	0.04	0.04
**6**	0.04	0.81	0.04	0.12
**7**	0.5	0.08	0.08	0.35
**8**	0.12	0.69	0.15	0.04
**9**	0.15	0.77	0.04	0.04
**10**	0.25	0.25	0.25	0.25
**11**	0.25	0.25	0.25	0.25
**12**	0.12	0.12	0.65	0.12
**13**	0.04	0.88	0.04	0.04
**14**	0.04	0.88	0.04	0.04
**15**	0.88	0.04	0.04	0.04
**16**	0.88	0.04	0.04	0.04
**17**	0.12	0.65	0.04	0.19
**18**	0.5	0.19	0.12	0.19
**19**	0.35	0.42	0.19	0.04
**20**	0.08	0.85	0.04	0.04
**21**	0.25	0.25	0.25	0.25
**22**	0.25	0.25	0.25	0.25
**23**	0.25	0.25	0.25	0.25
**24**	0.25	0.25	0.25	0.25
**25**	0.25	0.25	0.25	0.25
**26**	0.25	0.25	0.25	0.25
**27**	0.25	0.25	0.25	0.25
**28**	0.04	0.85	0.08	0.04
**29**	0.88	0.04	0.04	0.04
**30**	0.23	0.69	0.04	0.04
**31**	0.69	0.15	0.04	0.12
**32**	0.73	0.08	0.15	0.04
**33**	0.04	0.88	0.04	0.04

CpxR PSSM

Position	A	T	C	G
**1**	0	0	0	1
**2**	0.06	0.81	0.06	0.06
**3**	0.81	0.06	0.06	0.06
**4**	0.81	0.06	0.06	0.06
**5**	0.56	0.19	0.13	0.13
**6**	0.25	0.25	0.25	0.25
**7**	0.5	0.38	0.06	0.06
**8**	0.63	0.25	0.06	0.06
**9**	0.25	0.25	0.25	0.25
**10**	0.25	0.25	0.25	0.25
**11**	0.25	0.25	0.25	0.25
**12**	0	0	0	1
**13**	0.38	0.5	0.06	0.06
**14**	0.75	0.06	0.06	0.13
**15**	0.75	0.06	0.13	0.06
**16**	0.56	0.19	0.13	0.13
