# Supplementary material for: Genome-Scale Identification of Legionella pneumophila Effectors Using a Machine Learning Approach
Source: PLoS Pathog. 2009 Jul 10;5(7):e1000508. doi: 10.1371/journal.ppat.1000508 (PMC2701608; doi:10.1371/journal.ppat.1000508)
Supplement: Table S6 — (0.06 MB PDF) [file ppat.1000508.s007.pdf]

	lpg#	F-primer	R-primer	Enzymes used f		Resulting plasmid
1	lpg0080	lpg0080-EcoRI - GAGAGAATTCGATGAACAGATTAAAATTTTTTTCC	lpg0080-BamHI - GACCGGATCCTCATCGAGCATTAAACTC	EcoRI	BamHI	pZT-lpg0080-cyaA
2	lpg0090	Lpg0090-EcoRI - GACGAATTCAATGAAGCAGTTAATAAGTATAATAA	Lpg0090-BamHI - GACCGGATCCTACATTATACTTTCAAAGTTAC	EcoRI	BamHI	pZT-lpg0090-cyaA
3	lpg0096	lpg0096-EcoRI - GAGAGAATTCGATGCCTAAAGTTATTATTTTTACC	lpg0096-BamHI - GACCGGATCCAACGAAATAGAAATGCT	EcoRI	BamHI	pZT-lpg0096-cyaA
4	lpg0191	Lpg0191-BamHI - GACCGGATCCTATGATATCCCAAAAAGAAGCAC	Lpg0191-PstI - GACACTGCAGCTGATTATTTTAACTGAGGCTT	BamHI	PstI	pZT-lpg0191-cyaA
5	lpg0196	Lpg0196-XbaI - GAGGTCTAGAAATGAGTCTTGAAATCTATACCTGG	Lpg0196-PstI - GACGCTGCAGTTATGCTGGAACTCAACCG	XbaI	PstI	pMA-lpg0196-cyaA
6	lpg0240	Lpg0240-XbaI - GAGGTCTAGAAATGTGGTATCAAAATATAATGCG	Lpg0240-PstI - GACGCTGCAGGGACACTATATTGGTCCATT	XbaI	PstI	pZT-lpg0240-cyaA
7	lpg0285	Lpg0285-XbaI - GAGGTCTAGAAATGAGCAAAGAACGAATTAAGA	Lpg0285-PstI - GAGGCTGCAGATGAGCGCTCCATCAAAT	XbaI	PstI	pMA-lpg0285-cyaA
8	lpg0437	Lpg0437-EcoRI - GAGCGAATTCAATGCAAAACTTAGATGAGATTCT	Lpg0437-BamHI - GACCGGATCCTTTAGCACCCATAAACA	EcoRI	BamHI	pMA-lpg0437-cyaA
9	lpg0502	Lpg0502-XbaI - GAGGTCTAGAAATGAAAAAATCTATCCATCGATT	Lpg0502-PstI - GACGCTGCAGGTTCTGCTACTATACCAAAGGA	XbaI	PstI	pMA-lpg0502-cyaA
10	lpg0519	Lpg0519-BamHI - GACCGGATCCTATGCAAACCCAGAAAACCA	Lpg0519-Hind3 - GACGAAGCTTGTAGCACCTTTTAGCCTAAAAGG	BamHI	HindIII	pZT-lpg0519-cyaA
11	lpg0696	Lpg0696-EcoRI - GAGGGAATTCGATGAGAGATAAAATTATCACTGGG	Lpg0696-BamHI - GACCGGATCCTTGAAATTAAAATGGTAAGAA	EcoRI	BamHI	pZT-lpg0696-cyaA
12	lpg1101	Lpg1101-XbaI - GAGGTCTAGAGATGACTACGTCGGTGTCTTTTG	Lpg1101-PstI - GACGCTGCAGAGCTACCTCATTGCAAGCT	XbaI	PstI	pZT-lpg1101-cyaA
13	lpg1110	Lpg1110-EcoRI - GAGCGAATTCAATGGTCAGATTTTTCAAAGACA	Lpg1110-BamHI - GACCGGATCCCTTTTTAATAAGATAGCTCG	EcoRI	BamHI	pMA-lpg1110-cyaA
14	lpg1120	Lpg1120-EcoRI - GAGAGAATTCGATGACAACACCACTATTCCC	Lpg1120-BamHI - GACCGGATCCTTTGAATAGCTGGTAACTGT	EcoRI	BamHI	pZT-lpg1120-cyaA
15	lpg1121	Lpg1121-XbaI - GAGGTCTAGAGATGTCTAAACTAAGAGCATTGGAA	Lpg1121-PstI - GACGCTGCAGGAAAACTCCTGTTCAAA	XbaI	PstI	pZT-lpg1121-cyaA
16	lpg1145	Lpg1145-BamHI - GACCGGATCCTTTGGCTGATTCCAGATCATC	Lpg1145-Hind3 - GACGAAGCTTATAGACCAAAATCCGGATCG	BamHI	HindIII	pMA-lpg1145-cyaA
17	lpg1290	Lpg1290-XbaI - GAGGTCTAGAGATGCCTCAAATCCTAAATGC	Lpg1290-PstI - GAGCCTGCAGCTTTCTTTAAATAGGAAATCCTC	XbaI	PstI	pZT-lpg1290-cyaA
18	lpg1426	Lpg1426-EcoRI - GAGAGAATTCGATGACCCCACAGCACGTC	Lpg1426-BamHI - GACCGGATCCATGTGATGCAAACTCATACG	EcoRI	BamHI	pZT-lpg1426-cyaA
19	lpg1484	Lpg1484-EcoRI - ACGGAATTCGATGTCATCATTTAAACATTCTTGTG	Lpg1484-BamHI - GAGAGGATCCTCCGGGTTGTTCATGGTTAG	EcoRI	BamHI	pZT-lpg1484-cyaA
20	lpg1491	Lpg1491-EcoRI - GAGGGAATTCGATGAAACATTTAGCTGAATTAAAA	Lpg1491-BamHI - GACCGGATCCCCTTGATTAATAATATTACAA	EcoRI	BamHI	pZT-lpg1491-cyaA
21	lpg1496	Lpg1496-XbaI - GAGGTCTAGAAATGGTTACGAAAATAATTTGGG	Lpg1496-PstI - GACGCTGCAGCTCAAATACCATATTGATTTGCC	XbaI	PstI	pMA-lpg1496-cyaA
22	lpg1598	lpg1598-EcoRI - GAGAGAATTCGATGTGGTATGACACTCTATTGGAA	Lpg1598-XbaI - GACGTCTAGATTCATTAGTCCTCCTTGTTAAAGC	EcoRI	XbaI	pMA-lpg1598-cyaA
23	lpg1625	Lpg1625-EcoRI - GAGAGAATTCAATGCAAAATGAGAAAGAAGTAAA	Lpg1625-BamHI - GACCGGATCCATTGGAATGTATTGGTTTTAGC	EcoRI	BamHI	pZT-lpg1625-cyaA
24	lpg1702	Lpg1702-EcoRI - GAGGGAATTCGATGTCACTTGCTACTTATGATGTA	Lpg1702-BamHI - GACCGGATCCAGGCTGGAAATTAATGC	EcoRI	BamHI	pMA-lpg1702-cyaA
25	lpg1851	Lpg1851-EcoRI - GAGGGAATTCGATGTCATTTGAATTGGTCG	Lpg1851-BamHI - GACCGGATCCTGAGTGAAGAGGTGAGA	EcoRI	BamHI	pMA-lpg1851-cyaA
26	lpg1933	Lpg1933-EcoRI - AGGGAATTCGATGATTTATAAGATAACATTAACTG	Lpg1933-BamHI - GACCGGATCCTACGCAACAATAAATCAGA	EcoRI	BamHI	pMA-lpg1933-cyaA
27	lpg1947	Lpg1947-EcoRI - GAGAGAATTCAATGTTTTTTAAACGTGAAAAAA	Lpg1947-BamHI - GACCGGATCCATCAATTCAAATAATTTGAAT	EcoRI	BamHI	pZT-lpg1947-cyaA
28	lpg1949	lpg1949-EcoRI - GAGAGAATTCGATGGAAGACGAATCCAAAGTC	lpg1949-BamHI - GACTGGATCCGATGGAGACATATGAAAGA	EcoRI	BamHI	pMA-lpg1949-cyaA
29	lpg1969	Lpg1969-EcoRI - GAGGGAATTCGATGGCGTTATCAGAAATCATT	Lpg1969-BamHI - GACCGGATCCTGGCCTTCAAATTGAAAC	EcoRI	BamHI	pMA-lpg1969-cyaA
30	lpg2166	Lpg2166-XbaI - GAGGTCTAGAGATGATTTCAATAACAACTTATTGG	Lpg2166-PstI - GACGCTGCAGAACCTCGTATTTCAAACGG	XbaI	PstI	pZT-lpg2166-cyaA
31	lpg2206	Lpg2206-EcoRI - GAGCGAATTCAATGAACCGCATGGCACTTTT	Lpg2206-BamHI - GACCGGATCCTTATTCACAGCTAGCTGTGTGT	EcoRI	BamHI	pMA-lpg2206-cyaA
32	lpg2216	Lpg2216-EcoRI - GAGGGAATTCGATGAAGAGTAAAGAAGAACGATTT	Lpg2216-BamHI - GACCGGATCCCTGATTAATTAAACGGAC	EcoRI	BamHI	pMA-lpg2216-cyaA
33	lpg2248	Lpg2248-EcoRI - GAGGGAATTCGATGGCGAAGACAATTAAGGC	Lpg2248-BamHI - GACCGGATCCCATGAACAGTTACCTAAGG	EcoRI	BamHI	pZT-lpg2248-cyaA
34	lpg2328	Lpg2328-EcoRI - GAGGGAATTCGATGCCTGCTTTCAAAAAAGG	Lpg2328-BamHI - GACCGGATCCAGCGTTTAAAGACCAAATGC	EcoRI	BamHI	pZT-lpg2328-cyaA
35	lpg2406	Lpg2406-XbaI - GAGGTCTAGAAATGAAAAAGAAAGACGAGCAC	Lpg2406-PstI - GACACTGCAGCAATGTCTTAATTCATTGTCCC	XbaI	PstI	pZT-lpg2406-cyaA
36	lpg2411	Lpg2411-BamHI - GACGGATCCTATGTGGATTTTTAAAGATATTACCG	Lpg2411-PstI - GACGCTGCAGTTAAGTATACGATGGTCCCTTG	BamHI	PstI	pMA-lpg2411-cyaA
37	lpg2422	Lpg2422-XbaI - GAGGTCTAGAGATGAGCACATCAGAGAAAGAC	Lpg2422-PstI - GACGCTGCAGAATGGAACAGAGTTTAGTTC	XbaI	PstI	pZT-lpg2422-cyaA
38	lpg2433	Lpg2433-EcoRI - GAGGGAATTCGATGACGCCTTTGTTTCCAGA	Lpg2433-BamHI - GACCGGATCCTCTAGGGGAGTGATGTAAGGC	EcoRI	BamHI	pZT-lpg2433-cyaA
39	lpg2504	Lpg2504-EcoRI - CGGAATTCGATGACTAAAATATACTTATTAACTGC	Lpg2504-BamHI - GAGCGGATCCATATCAAAATACCAGTATCGATTC	EcoRI	BamHI	pZT-lpg2504-cyaA
40	lpg2507	lpg2507-EcoRI - GAGAGAATTCGATGCCTGAAGAGACGAGCAC	Lpg2507-BamHI - GACCGGATCCCGAGATTTCTTATGTTAAA	EcoRI	BamHI	pMA-lpg2507-cyaA
41	lpg2523	Lpg2523-XbaI - GAGGTCTAGAAATGAAAACCAAACTAAACAAATCA	Lpg2523-PstI - GACGCTGCAGACGTTATTTTATAGTTTAACCCACA	XbaI	PstI	pMA-lpg2523-cyaA
42	lpg2529	Lpg2529-XbaI - GAGGTCTAGAAATGTTTAATGTGGATAATAGTGGT	Lpg2529-PstI - GACACTGCAGCAAAAACAGCACCCTTA	XbaI	PstI	pZT-lpg2529-cyaA
43	lpg2603	Lpg2603-EcoRI - GAGGAATTCGATGCCTAAAATAAATAAAATTGTTA	Lpg2603-BamHI - GACCGGATCCAATGGACACGTCCTAAATGC	EcoRI	BamHI	pMA-lpg2603-cyaA
44	lpg2804	Lpg2804-EcoRI - GAGAGAATTCGGTGGGGCTTGAAAAAAAAT	Lpg2804-XbaI - GAGGTCTAGACGGCTGGATTTCAACCAAT	EcoRI	XbaI	pZT-lpg2804-cyaA
45	lpg2826	Lpg2826-XbaI - GAGGTCTAGAAATGCTTATGATCATGGCAAG	Lpg2826-PstI - GACGCTGCAGACGTTCGGTCATATCTTGT	XbaI	PstI	pZT-lpg2826-cyaA
